# Educating, training, and exercising for infectious disease control with emphasis on cross-border settings: an integrative review

**DOI:** 10.1186/s12992-020-00604-0

**Published:** 2020-09-03

**Authors:** Doret de Rooij, Evelien Belfroid, Christos Hadjichristodoulou, Varvara A. Mouchtouri, Jörg Raab, Aura Timen, for the EU HEALTHY GATEWAYS Joint Action consortium

**Affiliations:** 1 Centre for Infectious Disease Control, National Institute for Public Health and the Environment, Bilthoven, The Netherlands; 2 Athena Institute, VU University, Amsterdam, The Netherlands; 3 Department of Hygiene and Epidemiology, University of Thessaly, Thessaly, Greece; 4 Department of Organization Studies, School of Social and Behavioral Sciences, Tilburg University, Tilburg, The Netherlands

**Keywords:** Education, Training, Exercise, Infectious diseases, Cross-border, Training-of-trainers, Public health

## Abstract

**Introduction:**

Points of entry and other border regions educate, train, and exercise (ETEs) their staff to improve preparedness and response to cross-border health threats. However, no conclusive knowledge of these ETEs’ effectiveness exists. This study aimed to review the literature on ETEs in infectious disease control concerning their methods and effect, with an emphasis on cross-border settings and methods that enlarge ETEs’ reach.

**Methodology:**

We systematically searched for studies in the databases Embase, Medline, Web of Science, PsycInfo, ERIC, and Cinahl. After successively screening titles and abstracts, full-texts, and citations, 62 studies were included using in- and exclusion criteria. Data were extracted using a data-extraction form. Quality assessment was performed. We developed a theoretical framework based on which we analyzed the ETE context (target group, recruitment, autonomy, training needs), input (topic, trainers, development and quality of materials), process (design, duration, interval, goals), evaluation (pre-, post- follow-up tests), and outcome (reaction, learning, behavior, and system).

**Results:**

We found a limited number of published evaluations of ETEs in general (*n* = 62) and of cross-border settings (*n* = 5) in particular. The quality assessment resulted in seven ETE methodologies and 23 evaluations with a ‘good’ score. Both general studies and those in a cross-border setting contain a low-moderate detail level on context, input, and process. The evaluations were performed on reaction (*n* = 45), learning (n = 45), behavior (*n* = 9) and system (n = 4), mainly using pre- and post-tests (*n* = 22). Online learning methods have a high potential in enlarging the reach and are effective, particularly in combination with offline training. Training-of-trainer approaches are effective for learning; new ETEs were developed by 20–44% of participants until six months after the initial training.

**Conclusion:**

Our study reveals a limited number of publications on ETEs in infectious disease control. Studies provide few details on methodology, and use mainly short-term evaluations and low level outcomes. We call for more extensive, higher-level evaluation standards of ETEs, and an easy and sustainable way to exchange evaluations within the workforce of infectious disease control in cross-border settings. The theoretical framework developed in this study could guide future development and evaluation of ETEs in infectious disease control.

## Background

The risk of cross-border transmission of infectious disease pathogens increases with the rise in global travel of people and transfer of goods [1]. Travelers, goods or vectors infected in one place could transmit diseases to other travelers during their journey or infect the population in the country of destination. Locally, at points of entry (POEs) – airport, ports and ground-crossings – management of high numbers of infected or exposed travelers can be challenging and would have a significant economic impact. During the SARS and current COVID-19 pandemics, for example, entry- and exit screening was implemented at POEs worldwide [2, 3], as was contact tracing performed for hundreds of travelers [4]. Capacities and procedures for management of public health events at desginated POEs have been agreed by the WHO State Parties in the International Health Regulations (IHR) 2005 [5]. However, translating capacity into an appropriate, timely, and efficient response to cross-border spreading requires collaboration and communication between many disciplines, levels, and countries [6], and subsequently, ongoing efforts to stay prepared. To support many POEs at the same time, many partners, the World Health Organization, and the European Union have been organizing multi-national training programs and simulation exercises [7, 8].

Despite all these efforts, we currently have no insight into the different education, training, and exercises (ETEs) that are carried out on POEs and what their effect is. A literature review in 2017, studying training on infectious disease control, reported that the included studies contained insufficient detail on the methodologies of training and did not report any results [9]. To employ future efforts (time, costs, intentions) as efficient as possible, we integratively reviewed the available scientific literature [10, 11] to identify 1) the different ETE methodologies to train professionals in infectious disease management, 2) how these ETEs are evaluated and 3) what evidence is available for their effectiveness, with a particular attention on cross-border settings, such as POEs.

### The theoretical framework

**Fig. 1:**
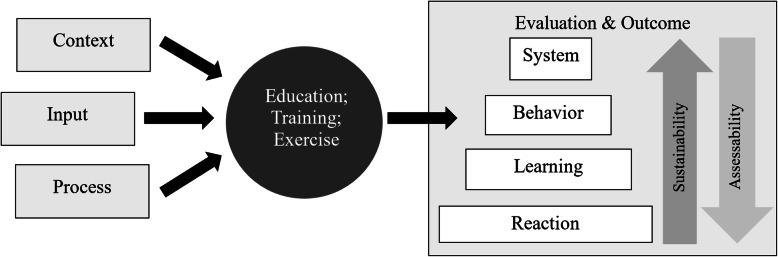
The context, input, and process affect the outcome of education, training and exercises. Outcome of education, training, or exercises can be evaluated at four levels (Kirkpatrick 1996). Lower levels are easier assessable, while higher levels show better sustainability of outcomes

To research the existing body of literature, we built a theoretical framework based on integrated theories and principles of effective teaching and learning. We combined the seminal *Kirkpatrick* [12, 13], *Input Process Outcome* [14] and *Context Input Reaction and Outcome* models [14, 15], the principles of adult learning [16, 17], the Self-Determination Theory on motivation [16], and techniques supporting sustainability [18]. In short, our framework states that ETEs rely on their context, input, and process and results in outcomes that can be evaluated at several points in time, and at four different levels (Fig. 1). The extensive theoretical background can be found in Additional file 1.

#### Context, input, and process

The context comprises the environment of the learner [13]. This context influences learning, and mainly the application and implementation of what is learned. An example is the participants’ ability to change existing practices in a larger system. Learning in a context that welcomes change stimulates learning and its application. Other contextual factors are the workload, the training needs, and the autonomy of learning and application for the specific target group. A context of specific interest is the cross-border setting, here defined as a setting with interaction between different nation-states, such as in border regions, points of entry, or other multi-country settings.

The input covers the external conditions of the ETE, such as the thoroughness and quality of the material development, participants’ prior knowledge, the ETE topic, and the facilitators’ experience [13, 14]. Regarding this last factor, the training-of-trainer (TOT) approach is of interest. In a TOT design, participants are raised as trainers or facilitators to deliver ETEs themselves, through which the reach of an ETE can be enlarged. However, the trainers’ quality should remain on a sufficient level.

The process comprises the implementation and design [13]. Either more classical designs are used, such as education based on presentations, training with workshops, or table-top exercises; or more innovative designs are used that enlarge an ETE’s reach or enhance realism. Other process factors are clarity of learning goals, interactivity and problem-based learning, and the duration and frequency of learning moments.

#### Evaluation & Outcome

According to our theoretical framework, the context, input, and process affect the effectiveness or outcome of an ETE. Three evaluation moments are distinguished; the pre-test right before the ETE, the post-test right after the ETE, and the follow-up test one to several months after the ETE. The pre-test is used to set the baseline for learning, the post-test is used to see the direct and short-term effect of the ETE, and the follow-up test assesses the sustainability of the effect over time. Also, control groups are required to exclude external effects. The ETE outcome can be evaluated at four levels: reaction, learning, behavior, system (Fig. 1) [12, 13]. The reaction level assesses participants’ satisfaction, either quantitatively or on content. The learning level assesses the improvement of knowledge, skills, or attitudes. Although knowledge and skills are best assessed using tests or demonstrations instead of self-assessments, for this study, both these objective and subjective measures are interpreted as learning. The behavior level assesses the change in individual working practice. Because objectively measuring behavioral change is often complicated and time-consuming, we include both objective and self-assessed change at this level. On the system-level, change is organizational. Examples are standard operating procedures, contingency plans, or the information or communication flow through an organization. While reaction and learning are more easily assessable, behavioral, or system change indicates higher sustainability of the outcome [12, 13]. Although lower levels are indispensable in motivating, monitoring and purposefully investing in the professionals that make up the public health system, the system level addresses the public health roles from a macro perspective. Outcomes on this level are therefore most relevant from a public health perspective.

### Education, training, and exercises

Based on our theory, education, training, and exercises are treated alike; these all aim at improving performance. Nevertheless, their differences are defined as follows: education is a process of individual learning in a general sense leaving several options for application available; training is a more practical and specified way of learning, also addressing practical aspects; exercises are a practical simulation of real practice.

## Methodology

### Literature search

To collect evaluations of ETEs in infectious disease control, we conducted a systematic, electronic search in the databases of Cinahl, Embase, Eric, Medline, PsycInfo, and Web of Science. The search period covered the period between the start of the databases (Cinahl: 1982; Embase: 1974; Eric: 1965; Medline 1946; PsycInfo: 1967; Web of Science: 1900) until 24 September 2018. We searched for a combination of “public health”, “infectious disease”, “cross-border”, “effectiveness”, “training” and their synonyms. The search strategy can be found in Additional file 2.

### Inclusion criteria

**Fig. 2:**
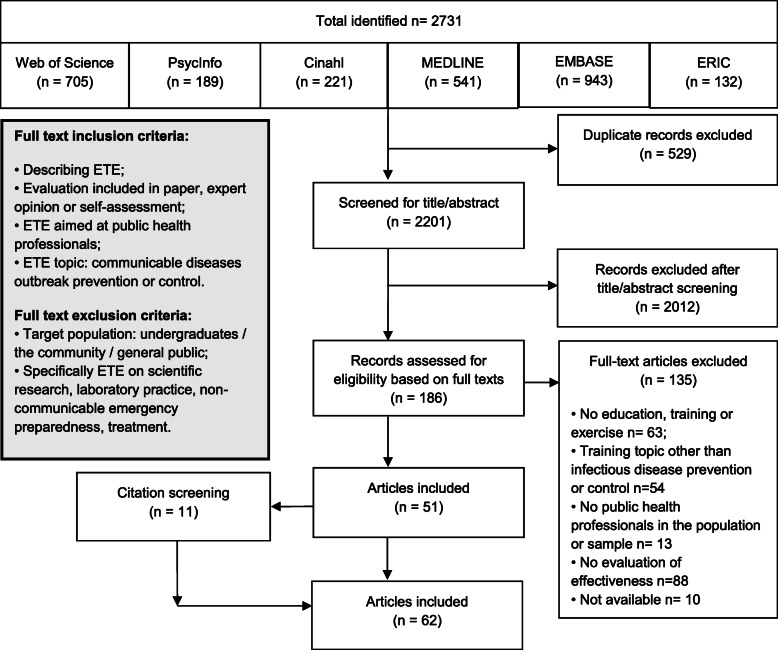
Flowchart of the systematic literature search

First, we screened titles and abstracts and included studies that described an evaluation of an ETE with a topic in infectious disease control from a public health perspective, or if compliance remained unsure. Subsequently, studies’ full texts were screened. Studies were included if an evaluation of the ETE was described in the paper and public health professionals, either on the local, regional, or national level, were among the target population. Studies were excluded if no public health professionals were included as participants or when the topic was restricted to research, a specific therapy, such as the use of anti-virals in a therapeutic setting, or laboratory practice. An overview of the in- and exclusion criteria is shown in Fig. 2. The reference lists of included studies were screened for additional relevant studies, using the same criteria. For both the abstract- and full-text screening, the first 25% of studies was screened independently by two authors (DdR, EB) and compared afterward. Any disagreements between the authors were discussed until consensus was reached, before continuing with the other 75% (DdR). In total, 62 studies could be included.

### Quality assessment

We assessed the quality of the ETE’s methodology and the quality of the evaluation for all studies. The first assessment was based on six questions from the Quality Standards in Education and Training Activities of the Youth Department of the Council of Europe 2016 [19], the second on six questions of the NICE Quality appraisal checklist for qualitative studies [20]. The quality assessment form can be found in Additional file 3. For both parts, a maximum of twelve points could be scored, leading to a bad, moderate or good score for tertiles. The first 25% of studies was scored independently by two authors (DdR, EB). After comparing and discussing the scores, one author continued (DdR).

### Data extraction and analysis

We performed an integrative review, inspired by the steps of Whittemore and Knafl [10, 11]. First, we designed a data extraction form based on the theoretical framework (Fig. 1). Then, we extracted data on variables of context, input, process, and outcome, as shown in Tables 1 and 2, along with basic study characteristics, such as the journal, publication year, country, and funding issues. We analyzed the context, input, process and the four outcome variables by describing their occurrence and variety. Sub-analyses were performed for studies in a cross-border setting or with a TOT approach. If many studies described one of the four outcome variables, results were subdivided according to education, training, and exercises, or even between classical and innovative study designs. If directions of outcomes highly differed, we compared for context, input, and process characteristics.

**Table 1:** Baseline characteristics of the included studies

***Study***		***Context***			***Input***			***Process***				***Evaluation***						
***Subgroup***	*1st Author, year, country, reference*	***Training Needs Assessed***	***Target group***	***Recruitment & Autonomy***	***Topic***	***Professional trainers*** *(content and/or didactics)*	***Development & Quality of the material***	***Intervention type***	***Duration & number of training moments***	***Train- & testable training objectives***	***Setting & Interaction***	***Pre-test*** *(RR response / total (%))*	***Post-test*** *(RR response / total (%))*	***Follow-up*** *(RR response / total (%))*	***Satisfaction*** *(evaluation method)*	***Learning*** *(evaluation method)*	***Behavior*** *(evaluation method)*	***System*** *(evaluation method)*
**–**	Ablah E., 2007, USA, [21]	–	All employees of local health departments	Invitations	Infectious disease outbreak response	–	–	Electronic, simulation exercise.	4 weeks	Goals stated at organizational level	Multi-county setting; a realistic time frame; participants received feedback to their responses.	RR 56/65 (86)	RR 48/65 (86)	–	–	Self-assessed skills	–	–
**–**	Ablah E., 2008, USA, [22]	–	All employees of local health departments	Invitations	Infectious disease outbreak response	–	–	Electronic, simulation exercise	4 weeks, 2–3 injects per day	Goals stated at organizational level	Multi-county setting; a realistic time frame; injects sent via a web-based system; participants received feedback to their responses.	–	RR -	–	Focus group	Focus-group: Self-assessed skills	–	–
**–**	Aiello A., 2011, Canada, [23]	Based on literature review & experience	Health professionals of all departments of a hospital	–	Resilience towards pandemics	2 members of the Psychosocial Pandemic Committee	-; Materials not tested.	Training	1 h	Implicitly named on organizational and individual level.	Information delivery and discussion.	RR 1250/1020 (82)	RR 1250/1020 (82)	–	Rating statements	Self-assessed skills	–	–
**–**	Alexander L.K., 2005, USA, [24]	–	Public health nurses	–	Communicable disease surveillance and response	–	–	Educational course	80 h	–	4 internet modules and one classroom module with presentations, discussion and technical consultation.	RR 55/80 (69)	RR 55/80 (69)	–	Rating statements	Knowledge test	–	–
**–**	Alexander L.K., 2008, USA, [25]	TNA among target group	Public health nurses	–	Communicable disease surveillance, −recognition, −outbreak investigation, and -control; new bioterrorist agents.	Professionals with strong teaching credentials	State partners provided content, academic partners provided curriculum development and distance-learning technical expertise.	Distance-education; face-to-face training and a table-top exercise	14 weeks	Clearly stated as competencies	Internet-modules with audio lecture, slides and additional readings; and 2 days face-to-face training with presentations and a tabletop on outbreak response	RR 177/156 (88)	RR 177/156 (88)	–	Rating statements	Self-assessed skills	–	–
**–**	Araz O.M., 2012, USA, [26]	–	University incident command, executive policy group, and emergency operations center.	–	Influenza pandemic preparedness	–	Collaboration between key university leadership, federal, state and local health officials, emergency response officials and key community stake holders.	Table top exercise and computer simulation model	1 day	Goals clearly stated	4 scenarios presented interactively and graphically; guided discussion; prompted decisionmaking; mixed-groups; feedback from a simulation model	–	RR -	–	Rating statements	–	–	–
**–**	Araz O.M. & Jehn M., 2013, USA, [27]	–	Local stakeholders: school administrators; local health officers; school nurses; first responders; parents; large community businesses	Invitations	pandemic planning	–	–	Table top exercise	1 day	Goals clearly stated	Mixed-groups; three scenarios; guided discussion of predefined questions and response in a group-setting; feedback from a simulation model.	RR 177/156 (88)	RR 177/156 (88)	–	–	Self-assessed skills	–	–
**–**	Atack L., 2008, Canada, [28]	–	Healthcare professionals, educators, coordinators and others	–	Infection prevention and control	n/a	A model of continuing education (Cervero (1985)) describing the complexity of knowledge transfer. Content developed by ICP experts	Online course	–	–	3 modules using text, graphs, videos, quizzes and games	RR 67/76 (88)	RR 67/76 (88)	–	Rating statements	Knowledge test	Naming examples of changed behavior	–
**–**	Atlas R.M., 2005, USA, [29]	–	Medical students, practicing physicians and other healthcare professionals	–	bioterrorism	–	–	–	–	Several goals stated as examples	Using standardized patients and patient simulators	–	RR -	–	–	Self-assessed attitude	Self-assessed behavior	–
**–**	Baldwin K., 2005, USA, [30]	In target group and on organizational level.	Public health personnel	–	multidisciplinary response to bioterrorism	n/a	Followed the template developed by Columbia University School of Nursing & College of Health Policy (2002); Collaboration between public health nursing administration and faculty at a university school of nursing.	4 e-modules (series)	–	–	On the intranet of a public health department	–	RR 15/15 (100)	–	Open ended question	Knowledge test	–	–
CBS	Bazeyo K.M., 2015, Uganda, [31]	–	Health workers, immigration officers, customs and media.	Selection	Ebola surveillance, preparedness and response	Professionals with experience in training delivery and on content.	–	Training	5 days	Stated specific per discipline, formulated as topics	6 border districts in Uganda; using mixed groups and participatory methods.	RR 330/− (−)	RR 330/− (−)	–	–	Knowledge tests	–	–
**–**	Becker K.M., 2012, Ghana, Uganda, Nigeria, USA, [32]	–	Midlevel public health leaders and frontline public health surveillance workers	Recruitment	Surveillance and response systems; laboratory networks	Universities as host institutions	Ministries of agriculture and veterinary schools together with ministries of health and public health training institutions during planning, development, and implementation	In-service postgraduate program	2 years	competency-based courses, not shown. General study goal stated on regional level.	Mix of 25–35% classroom and 65–76% field-based training; interaction between public health and veterinary professionals.	–	RR 43/43 (100)	–	–	–	–	Summing up organizational achievements in two years
TOT	Berrian A.M., 2018, USA, [33]	–	Environmental monitor residents	–	Professional skills & one health	Study investigators.	Based on inquiries from a biosecurity project, supported by theoretical underpinnings in constructivist learning and social cognitive theory; pilot tested first.	Training-of-trainers (unclear methods)	4 weeks	Objectives clearly stated.	‘workshops with unknown methods; Training delivery directly after the training.	RR 10/10 (100)	RR 10/10 (100)	–	–	Knowledge test; Self-assessed skills	–	–
**–**	Biddinger P.D., 2010, USA, [34]	–	Health-care organizations (30%), health departments (17%), emergency management agencies (12%), fire departments (8%), law enforcement (6%), schools (6%), volunteer organizations (6%), town administration (3%), federal government (3%), community health centers (2%), and other (7%)	–	Public health preparedness	–	Conform the Homeland Security Exercise and Evaluation Program (HSEEP) and consistent with the principles of the National Incident Management System.	38 guided, PHEP table-top, functional, drills, and full scale simulation exercises.	–	Generally stated	Interactive, multi-disciplinary, regional, and mixed-group methods. “Realistic to the greatest extent possible”	Tabletops RR 1145/5892 (19.4);	Tabletops RR 1145/5892 (19.4)	–	Rating statements	Knowledge tests; Self-assessed attitude	–	–
**–**	Cathcart L.A., 2018, USA, [35]	–	All new staff at a CDC State Coordination Task Force	Mandatory	Zika virus response	‘4 instructors’	The Division of State and Local Readiness Applied Learning and Development Team (ALDT) at the Centers for Disease Control and Prevention (CDC); according to a just-in-time-training template.	Training	< 2 days	Clearly stated as competencies	–	RR 120/120 (100)	RR 120/120 (100)	RR 59/120 (49)	Rating statement	Self-assessed skills	Self-assessed behavior	–
**–**	Chandler T., 2008, USA, [36]	–	Employees of local health departments	–	Basic emergency preparedness training	Local supervisors	The CU-CPHP’s curriculum development; considering the options in blended learning literature.	Online distance learning program; on-site agency-specific program.	-; + 2 days on-site	Competency-based	Nationwide, on-site trainings in interaction with local organization & supervisor.	RR 817/> 817 (−)	RR 817/> 817 (−)	–	–	Knowledge test; Self-assessed knowledge; Skills test	Supervisor’s evaluation	–
**–**	Chiu M., 2011, USA, [37]	–	Public health nurses	–	Disaster surge	–	Competency-based, relies on adult learning principles	Online and in-class training	50 h in 12 months	Competency based	12 self-learning, online modules and one face-to-face interactive classroom session.	RR 41–54/182 (23–30)	RR 41–54/182 (23–30)	–	–	Self-assessed skills	–	–
**–**	Craig A.T., 2007, Australia, [38]	–	Emergency departments’ (ED) and regional health departments’ staff	“required”	Regional health departments’ pandemic early response	–	–	Simulation exercise	3.5 h	Stated on an organizational level	Very realistic. Mimicking patients suspected for influenza admitted to EDs.	RR -	RR -	–	Rating statement	–	–	Self-assessed system performance
**–**	Dausey D.J., 2007, USA, [39]	–	State and local health departments	–	Emergency preparedness for manmade and naturally occurring biological threats	–	Materials tested several times	31 Table-top exercises.	2–8 h	–	Limited- active involvement of the facilitator. Shared common elements: evolving hypothetical scenarios, facilitated group discussions, collective decision making.	–	RR 513/− (−)	–	Exercise debriefing; internal team discussion; open ended evaluating questions; after action reports.	–	–	–
CBS	Dausey D.J., 2014, USA, [40]	Training objectives were identified in previous exercises	**–**	Selection by the exercise planning team.	–	Experienced in training delivery and on content.	Experienced team, based on the “Day After” methodology	12 Table-top exercise	1.5–4.5 h	Objectives remain implicit - not stated.	Outside the USA (a.o. Southeast Asia, Middle East, East Africa), in a multi-sectorial, sub-national, national and sub-regional setting. All exercises included a presentation of the scenario, table-top exercise, guided discussion on 3–6 topics, decision making, and a debriefing.	–	RR −/558 (−) participants	RR −/137 (−) observers	Satisfaction & methodology	Self-assessed knowledge; Self-assessed attitude	Self-assessed behavior: Reporting on ministry level whether learning had changed behavior	–
**–**	Dickmann P., 2016, Hong Kong, Poland, Sweden, Switzerland, UK, [41]	–	Public health and communication experts working at ECDC and the Commission of the European Union	–	Risk communication on preventions and control of communicable disease threats	–	Extensive theoretical background on risk communication; team of risk communication experts convened by ECDC.	Training program	2 days	Clearly stated	Input of participants for case-studies reflection sessions, discussions, exploration, testing, working on scenarios, feedback from others in small working groups	RR 15/15 (100)	RR 15/15 (100)	–	Rating statements	Self-assessed knowledge; Self-assessed attitude	–	–
CBS	El-Bahnasawy M.M., 2014, Egypt, [42]	**–**	Young, military nursing staff, mainly unexperienced on the topic	**–**	Infectious disease disasters at the Eastern Egyptian Border.	**–**	**–**	Training	–	–	In the Egyptian border region; −	RR 125/− (−)	RR 125/− (−)	RR – (−)	Satisfaction & methodology	Knowledge tests	–	–
TOT	Faass J., 2013, USA, [43]	Yes	Transit personnel	Voluntary	Training skills & H1N1 prevention in the transit industry	–	Development based on previous trainings, new research and expert consultations.	Training	Half a day	–	Presentation; 1,5 h webinar or train-the-trainer session; resource book and pamphlet	–	RR 120/231 (52)	–	Satisfaction	Self-assessed attitude	–	–
**–**	Fowkes V., 2007, USA, [44]	Educational needs assessment in each area.	Health professionals practicing in medically underserved areas.	–	Public health emergency preparedness in medically underserved areas	Multi-disciplinary group of a faculty was trained to conduct the educational sessions; trainers were pharmacists, physicians, administrators, family physicians, and other.	Based on the needs assessment, the guidelines for core competencies (U.S. CDC), national guidelines from the National Incident Management System; expertise of medical directors, preparedness experts, program director and evaluator with academic experience in medical education.	Training	4–6 times 1 h	Stated as competencies.	multi-disciplinary, face-to-face trainings using presentations and case-studies.	–	RR > 6000/9537 (> 62.9)	–	Open ended question; Rating statement	Self-assessed knowledge; Self-assessed attitude	–	–
**–**	Fowkes V., 2010, USA, [45]	–	Health professionals	Self-selection & recruitment.	Development and application of emergency plans	Local health professionals with interest and expertise in emergency preparedness.	“Based on California’s guidelines for community clinic emergency plans and resources from the Hospital Bioterrorism Preparedness Program; the cal-PEN medical director developed a scenario for two exercises”	90 table-tops	–	Stated as competencies	On-site locations in 18 counties; role play in disaster scenario, enacted, evaluated.	RR 1176/1496 (78.6)	RR 1176/1496 (78.6)	RR 1176/1496	Rating statements	Self-assessed knowledge; Observed skills		Check of operation plans in quarterly reports; assessment of the departments’ emergency plans; reviewed AARs completed by the exercise groups;
**–**	Gershon R.R., 2010, USA, [46]	–	Emergency medical services (EMS) personnel	Mandatory, department-sponsored.	Pandemic preparedness (routes of transmission, PPE use, control practices, seasonal vaccination)	Trained EMS station officers	–	Training	30 min	Stated	Small group-setting with presentation, demonstration and a drill.	RR 129/− (−)	RR 129/− (−)	–	Rating statements	Knowledge test; Self-assessed knowledge	–	–
TOT	Grillo M., 2017, [47]	–	Military, medical doctors from developing countries.	–	Military, international HIV	–	Based on behavioral, social and cognitive learning, and international recommendations; in collaboration with military organizations.	Training	4 weeks	–	Clinical training, discussions, lectures	RR 136/136 (100)	RR 136/136 (100)	–	–	Knowledge test	–	–
**–**	Hegle J., 2011, USA, [48]	–	Federal, state and local health departments.		Surveillance	–	–	Different exercises	–	Implicitly stated	On-site, with own colleagues; tabletops; seminars; functional exercises; workshops	–	RR −/682 (−)	–	–	–	–	AAR:Observation by at least 2 researchers using an observation guide; semi-structured interviews with exercise leaders; review of planning and exercise materials
**–**	Hoeppner M.M., 2010, USA, [49]	A learning needs assessment	Public health professionals at a university	Application	Emergency preparedness	University staff	Development by University of Minnesota School of Public Health based on learning needs; grounded in an educational model proposed by Benner.	Education curriculum	Months-years	Stated as competencies	“courses”	–	–	RR 244/387 (63)	Rating statements	Self-assessed skills; Self-assessed attitude	Self-assessed behavior	–
**–**	Horney J.A., 2005, USA, [50]	–	Epidemiologist, public health nurses, health educators, health service manager/ -administrator/ -directors, environmental health employees.	Free online	Public health preparedness	Regional PH faculty and guest lecturers from PH schools, medicine, pharmacy, and government.	Developed by the lecturers and in line with the competencies	E-modules	0.5–1 h	Modules based on core competencies	Lectures and slides	–	RR 416/3030 (14)	–	Rating statements	Knowledge test; Self-assessed attitude	–	–
**–**	Hueston W.D., 2008, USA, [51]	–	Public health and veterinary medicine schools	University students	Population health, primary prevention, disease outbreaks	University staff	Universities	Joint degree program	2 years	–	Classroom, laboratory, and clinical education.	–	–	RR -	Sharing lessons learned.	–	–	–
**–**	Johnson Y.J., 2009, USA, [52]	–	Central, regional and local PH professionals, emergency management-, agricultural-, police-, and industry professionals.	–	Food-borne terrorism outbreak	–	–	Functional simulation exercise	2 days	Clearly stated, on organizational and individual level	Mixed groups of health- and non-health responders. Briefing, injects and interaction via a blog website	–	RR -	–	Rating statements	Self-reported skills	–	–
**–**	Kohn S., 2010, USA, [53]	Named as relevant; not performed	Local public health departments	–	Incident management system use	“selected trainers” of the John Hopkins-Center for Public Health Preparedness	By the Johns Hopkins Center for Public Health Preparedness very extensively described: out of quite sec NIMS, content was made PH specific.	training	3–7 h	From an organizational perspective.	Face-to-face modules with presentations, slides, open book exam, interactive lecture materials, specific and attractive for the target group	–	RR 213/− (−)	–	Rating statements Informal conversations with participants and trainers	–	–	–
TOT	Livet M. 2005, USA, [54]	–	Public health staff and community partners	–	Development & implementation of a table-top exercise & emergency preparedness for local community capacity	Academic experts & sponsors of the program.	–	Training-of-trainers	Three times a 2-day session.	Testable & trainable goals	1) Presentations, interaction, exercises, motivational presentations; 2) lectures, case-studies, discussions; 2–3) delivery of own TTX; 3) active presenting and discussion.	RR 67–70/80 (84–88)	RR 67–70/80 (84–88)	–	Self-assessed networking/ relationship building	Self-assessed competence	–	–
**–**	Macario E., 2007, USA, [55]	On an organizational level; not among participants	Public health nurses and other health professionals	–	Pandemic influenza	Presenters: CDHS Communicable Disease Control and Immunization Branch public health medical officers and laboratory research scientists, public affairs professionals. Local facilitator of the tabletop.	California Department of Health Services and the California Distance Learning Health Network	Table-top exercise	3,5 h divided over two sessions at the same day	Stated as SMART goals derived from competencies	Online lectures and tabletop exercise at the same day; on-site and with local partners.	–	RR: broadcast 821/25000 (3.3) RR: tabletop 164/− (−) RR knowledge test: 735/25000 (2.9) RR teleconference: 21/− (−)	–	Rating statements; Telecall interviews after table-top	Knowledge test; Telecall interviews after table-top on skills and confidence	–	–
CBS	Martin G., 2018, Ireland, [56]	**–**	Airport- &PH personnel, fire officers, police, health service responders	**–**	Response to a plane with MERS-suspection on board.	**–**	Exercise material reflected the WHO Simulation Guide.	Simulation exercise		Organizational level	Several areas on a local airport available: operation control center, passenger reception, real plane and runaway.	–	RR - /> 200 observers (−)	–	Satisfaction & Methodology	–	–	–
**–**	Mitka M., 2003, USA, [57]	–	National, state and local health and safety officials	–	Bioterrorism event in the metro	–	–	Simulation exercise	5 days	Implicitly stated	City-wide exercise, on-site and with many partners	–	RR -	–	Participants’ comments	–	–	–
**–**	Morris J.G., 2012, USA, [58]	–	4 federal agencies, 9 state agencies, 6 universities, 1 nonprofit organization, and 1 private corporation	Invited	Foodborne toxoplasmosis outbreak on college campuses	–	Ad hoc planning committee within a regional partnership of universities, public health agencies, affiliates, and foundations dedicated to combating biologic threats	Tabletop exercise	2 days	Stated as competencies	5 modules representing phases of the outbreak, multimedia depiction of simulated conditions, guided small-group discussions, plenary discussions.	–	RR 22/− (−)	–	Rating statements	Self-assessed knowledge	–	–
**–**	Olson D., 2008, USA, [59]	Yes, using a Delphi method	Current and future public health workers	‘admitted’	bioterrorism and emergency readiness at a school of PH	–	UMNSPH’s lifelong-learning model based on the Dreyfus model (Benner), and Spross & Lawson	Education curriculum	17 h	Competency-based curriculum	–	–	RR −/1680 (−)	RR -	Testimonials	Self-assessed knowledge (testimonials)	Self-assessed behavior (testimonials)	–
TOT	Orfaly R.A., Frances J.C., 2005, USA, [60]	Community needs assessment	–	Recruited based on their interest and experience in capacity building and public speaking.	Delivery of educational programs & public health preparedness	Public health directors for training.	Based on a community needs assessment, and adult learning principles.	Training		Objectives generally stated.	2-days training in training and monthly lectures thereafter on preparedness. Participants had to perform 3 own trainings of which 1 < 90 days after	–	–	RR 21/21 (100)	Satisfaction through interviews	–	# Delivered trainings & # participants	–
**–**	Orfaly R.A., Biddinger P.D., 2005a, USA, [61]	–	Students of the Master of Public Health	Self-registered	Bioterrorism preparedness and response	Practicing emergency physicians +experts in disaster medicine or emergency preparedness and response.	Based upon previously existing course, further adapted to serve as training.	Course in the master of PH	7-weeks: 30 in class hours	Core competencies stated during evaluation of the course, not as course goals	Series of lectures (30 h) and a 2-day tabletop exercise	–	RR 24/24 (100)	–	Rating statements	–	–	–
TOT	Otto J.L., 2010, USA, [62]	–	Military PH emergency officers	–	Influenza response	Facilitators were “trained”.	Based on organizational needs and policy, not tested.	Table-top exercises	–	Training objectives clearly stated.	Table-tops with prepared questions, guided discussion, and a hot wash. Own table-top was expected afterwards	–	RR 65/85 (76)	RR 50/85 (59)	Satisfaction & methodology through Likert scale questions and open questions	–	Self-assessed new/revised planning; whether exercises were performed	–
**–**	Peddecord K.M., 2007, USA, [63]	–	PH professionals	Freely available online	Mass vaccination service	–	Produced by department of health services, a distance learning network and the center for disease control	Online training	90 min	–	90 min online lecture, more specific methods unknown	RR 520/> 1658 (< 31)	RR 520/> 1658 (< 31)	RR 291/> 1658 (< 18)	–	Knowledge test; Rating statements on attitude	Self-assessment of behavior with open ended questions	–
**–**	Potter M.A., 2005, USA, [64]	Yes, unknown method	Public health workforce of several counties	Recruited	Leadership in emergency preparedness and counter terrorism	–	Based on an existing leadership curriculum	Training curriculum	A Year	Stated on the organizational level	Three conferences and a real-life project	–	RR 28/28(100)	–	Rating statements	Self-assessed knowledge & skills	–	–
**–**	Quiram B.J., 2005, USA, [65]	–	Physicians, veterinarians, epidemiologists, nurses, law enforcement personnel, emergency medical technicians, hospital safety officers, port authority personnel, bioterrorism planners and coordinators. 50% serves rural populations	–	Emergency preparedness & response	Experts on the topic from a variety of organizations.	School of Rural Public health at Texas A&M University, CDC.	Training	3 modules of 4,5; 2; 2 days, spread over several weeks	Stated as competencies or concrete SMART tasks.	Multi-methods, including presentations, simulation, table-top exercise, technical consultation and discussion	RR −/167 (−)	RR −/167 (−)	–	–	Knowledge test	–	–
**–**	Qureshi K.A., 2004, USA, [66]	–	PH nurses	Recruited	Emergency preparedness	Columbia University faculty members and School Health Program staff, senior leadership from the NYC–DOHMH	Developed in consultation with the NYC–DOHMH School Health Program administration; based on CDC’s Emergency Preparedness Core Competencies for All Public Health Workers.	Training	4 h	Based on the basic public health emergency preparedness competencies	Presentations and readables	RR 678/764 (89)	RR 678/764 (89)	RR 230/764 (30)	Rating statements	Knowledge tests; Self-assessed knowledge; Self-assessed attitude	–	–
**–**	Rega P.P., 2013, USA, [67]	–	Students in the master of PH	–	Pandemic preparedness and response	–	–	Education & a table-top exercise	Semester	–	1) education, 2) audio materials mimicking a growing pandemic. 3) Tabletop exercise, groups representing counties, group response. Second table-top was adapted based on feedback.	RR -	RR -	–	Rating statements	Self-assessed knowledge	–	–
CBS	Richter J., 2005, USA, [68]	**–**	**–**	Recruited per e-mail and telephone.	Bioterrorism	Selected on experience & responsibility.	Newly developed by parties experienced on content	Table-top exercise	2 days	Training goals: interagency networking while assessing their training and research needs.	At a cruise ship. Using presentations, guided-group discussion, small-group guided discussions, plenary presentations, networking.	–	RR 32/50 (64)	–	Satisfaction, Methodology	Self-assessed knowledge	–	–
**–**	Rottman S.J., 2005, USA, [69]	Inquiry of agency’s disaster plan and local emergency management policies are inquired.	All levels of health department personnel.	–	Disaster preparedness & response	The Center for Public Health and Disasters as organizing company.	Applied preparations per location: an interview at the department and profiling the community and the environment.	Training & exercise	2 days	Clearly stated competencies are used	Interactive, scenario-based training sessions, Location & agency-specific training and 4 table-top exercises	RR 403/− (−)	RR 403/− (−)	–	–	Knowledge test; Self-reported knowledge	–	–
**–**	Sandstrom B.E., 2014, Sweden, [70]	–	Emergency board personnel from a wide range of functions, PH personnel	–	CBRN emergencies	Adequate emergency management experience, fully prepared and comfortable in their position	Iterative process of application and adaptation to a local homogeneous, national mixed and international mixed setting.	Table-top exercise	–	–	Using exercise cards to walk participant through the scenario. Led to different possible outcomes of the scenario.	–	RR n/a	–	Observation of the exercises; Evaluation seminars	–	–	–
**–**	Sarpy S.A., 2005, USA, [71]	A needs analysis	Representatives from the Arkansas department of health and external partner agencies	–	Response to a SARS event	Local physician with an advanced degree in PH, expertise in facilitating small group discussion, knowledge of the local healthcare system. The expert in SARS presented the pre-tabletop lecture, is an international authority on SARS, with hands-on experience.	According to the SCCPHP training systems model; Centers for Disease Control and Prevention core competencies for emergency preparedness and response and input from practice partners	Tabletop exercise	Half a day	Objectives based on competencies which were identified in the needs assessment	1) Lecture; 2) 3.5-h tabletop in 7–10 p groups where 3 scenarios were discussed (first individually, then discussion of answers, group decision, plenary presentation and discussion). Tricks to enhance realism: first ambiguity in case, participants became infected, authentic contextual factors integrated, 30-day time frame, info on a day-to-day basis.	RR 49/49 (100)	RR 44/49 (90)	–	Rating statements; Open ended questions	Self-assessed skills; Self-assessed knowledge; Self-assessed attitude	–	–
**–**	Savoia E., 2009, USA, [72]	–	Local-, regional-, and state-level professionals from a variety of disciplines such as public health, law, health care, public safety, and emergency management.	–	Legal preparedness	An expert knowledgeable on PH infrastructure of the geographical area being tested	Using program guidance provided by the Association of State and Territorial Health Officials	Tabletop exercise	–	Stated as competencies within the text	Presentations, three exercise modules, guided small-group discussions, mixed groups from same/ neighboring communities	RR 56/89 (63)	RR 56/89 (63)	–	–	Knowledge test; Self-assessed attitude	–	–
**–**	Savoia E., 2013, USA, [73]	–	Public health officials and emergency responders with experience in emergency preparedness exercises	A convenience sample	PHEP&R	–	Opinions from 61 public health officials and emergency responders were systematically gathered and analyzed	Consensus method on the use of exercises and AARs.	–	Clearly stated questions	Consensus method	–	RR -	–	Group discussion developing lists of recommendations	–	–	–
TOT	Soeters H.M., 2018, Guinea & USA, [74]	–	Infection prevention and control trainers; frontline healthcare workers at health centers.	–	Conducting needs assessments & Regional infection prevention;	–	PH ministry, WHO and CDC.	Training	3–4 days training-of-trainers; subsequent 2 days training delivery	Training objectives stated	During the Ebola epidemic, at an health center, training delivery directly after the TOT. Program: 55% didactic methods, 45% hands-on training with practice, demonstration and technical assistance.	RR 1625/1625 (100)	RR 1625/1625 (100)	–	–	Knowledge test; demonstration of skills	–	–
**–**	Taylor J.L., 2005, USA, [75]	–	A broad selection of public health staff and emergency services	Recruited during two conferences	Pandemic influenza preparedness	–	Collaborative effort between DHMH, the Maryland Partnership for Prevention, and a group of outside consultants.	Tabletop exercise	4 h	Stated on an organizational level.	1) two introductory presentations, 2) 9 scripts on 1 outbreak were presented, 3) individual response 4) group discussion 5) joint action. Each participant was allowed to bring up to two additional experts for consultation on an as-needed basis.	–	RR 69/150 (46)	–	–	Self-assessed attitude by comments and written evaluations	–	–
**–**	Umble K.E., 2000, USA, [76]	–	Trained in nursing, clinical or managerial duties, and worked for a state, city, or county public health agency	–	Vaccine-preventable diseases	–	With the help of instructional and graphic designers	Traditional classroom vs. distance education	14 h	Stated as a single course goal	–	RR 196/470 (41.7) for **classroom** RR 116/251 (46.2) for **broadcast**	RR 196/470 (41.7) for **classroom** RR 116/251 (46.2) for **broadcast**	RR 196/470 (41.7) for **classroom** RR 116/251 (46.2) for **broadcast**	–	Knowledge test; Self-assessed attitude; Self-assessed skills;	–	–
**–**	Waltz E.C., 2010, USA, [77]	–	PH professionals in New York State	Differed among and within used methodologies	Preparedness training	University of Arkansas center for public health preparedness staff members	–	3 education technologies: audience response systems, satellite broadcast and interactive web-based continuing education courses in public health.	–	–	Audience response systems, satellite broadcast and interactive web-based continuing education courses in public health.	–	ARS RR 93/93 (100); Satellite broadcast RR none; Web-based education RR 20.000/44.000 (48)	–	Survey; # views	–	–	–
**–**	Wang C., 2008, China, [78]	On individual and organizational level; TNA is part of the public health leadership model referred to.	Public health leaders	–	Emergency response	Selected on their expertise in the field of PH emergency response, related training programs and involvement in continuous consultations on health service programs.	Training developed according to the public health leadership model on development, delivering and evaluating training.	Mixed-methods leadership training	14 days	Clearly stated as competencies	Mixed-methods	RR 41/43 (95)	RR 41/43 (95)	RR 41/43 (95)	Rating statements	Knowledge test; Self-assessed skills	–	–
**–**	Wang C., 2008a, China, [79]	–	Public health staff at centers for disease control and prevention in 18 cities	–	Emergency preparedness	Based on their expertise, from the MOH, WHO, Chinese CDC, Health Department of Hubei Province, Fudan University, Wuhan University and Huazhong University of Science and Technology	Based on the aims	Training	–	Aims designed by experts, based on competencies, stated as topics	Case-studies, workshops, tutorials, seminars, group discussions, role playing, drilling and fieldwork. Least used method was formal lecture.	RR 76/78 (97)	RR 76/78 (97)	RR -	Rating statements	Knowledge test; Self-assessed skills	–	–
**–**	Wang C., 2010, China, [80]	Yes	Public health staff in rural centers for disease control	–	Emergency preparedness	From MOH, WHO, Chinese CDC, Wuhan University and Huazhong University of Science and Technology. selected based on expertise in the field of Public health emergency response	According to an integrated instructional design system model (Fig. 1), which emphasizes the major components of instructional design, including assessing, designing, delivering and evaluating training.	Training	–	Using core competencies,	Case-studies, workshops, tutorials, seminars, group discussions, role playing, drilling and fieldwork. Least used method was formal lecture.	RR 226/237 (95)	RR 226/237 (95)	RR -	Rating statements	Knowledge test; Self-assessed skills	–	–
**–**	Yamada S., 2007, Hawaii, [81]	–	Physicians, nurses, public health workers, hospital administrators, lab workers, radiology technicians, medical records clerks, pharmacy workers, cancer registrars and dental assistant, and other.	–	Response to unknown agents	Trained in-country personnel on PBL in PBL tutoring skills, education and training.	At the University of Hawai‘i, by the Pacific Bioterrorism Curriculum Development Project, Based on the principles of and experience with PBL, community-based, and interdisciplinary training.	Education	Several meetings/ working groups	–	Interdisciplinary problem-based, guided discussion of a case, lists of problem, discovery learning in groups, presentations and discussion.	–	RR 85/− (−)	–	Rating statements; Interviews with participants	–	–	–
**–**	Yellowlees P., 2007, USA, [82]	–	State and county health officials	–	Mass prophylaxis delivery	n/a	Based on an assessment of normal work flows and surroundings, by authors and the help of a graphical artist	Virtual reality training	2 h	-, each participant had their own objectives	Virtual reality pilot: the SecondLife game environment with participants for introduction/guided tour and taking up virtual tasks like reception, screening, examination, and dispensing.	–	RR 13/25 (52)	–	Rating statements; Open ended questions	–	–	–

Variables include characteristics of context, input, process and eveluation. RR = response rate; TOT = training-of-trainers; CBS = cross-border setting; − = no information available; EMS = emergency medical service, PPE = personal protective equipment; PH = public health; # = ‘the number of’

11

**Table 2:** Results

	***Study*** ***(1st Author, year, country, titel)***	***Evaluation method***	***Pre-test***	***Post-test***	***Follow-up***
	Ablah E., 2007, USA, [21]	Online questionnaire with 5-point-Likert-scale answer options	RR 56/65: 86% Self-assessed skills: - to identify the need for and implement surge capacity: 43% good / excellent - Participate in a coordinated response: 32% good / excellent - To implement your risk communication skill set: 12% - To identify and locate your agency infectious disease resrouces: 49%	RR 48/65: 74% Self-assessed skills: - ability to identify the need for and implement surge capacity: 74% good / excellent (*p* = 0.003) - Participation in a coordinated response: 60% good / excellent (*p* = 0.017) - To implement your risk communication skill set: 25% (not sign) - To identify and locate your agency infectious disease resrouces: 60% (not sign)	–
	Ablah E., 2008, USA, [22]	< 4 weeks after the exercise, focus groups were held, transcribed and main themes identified	-	RR unknown Self-reported skills: improvements in surge capacity, coordination between counties, risk communication, and awareness of protocols and procedures; better able to (be) support (ed), they see necessity of a coordinator, effective relation building. Satisfaction/ training content/methodology: The exercise format was liked.	-
	Aiello A., 2011, Canada, [23]	Retrospective pre-test & post-test: questionnaire with 5-point-Likert-scale answer options	RR 1250/1020 (82) Self-assessed skills: 35% felt prepared to deal confidently with the situation during a pandemic	RR 1250/1020 (82) Response Rate Self-assessed skills: 76% felt prepared to deal confidently with the situation during a pandemic. (*p* = 0.0020). Satisfaction “a high proportion” thought the training relevant for work- & personal life, useful, helpful and informative.	-
	Alexander L.K., 2005, USA, [24]	Pre-test and post-test on knowledge with MCP questions after each online module; course evaluation after 5th module with 4-point-likert-scale and open answer options.	55/80 (69) Knowledge results not available	55/80 (69) Knowledge results not available Satisfaction: 100% recommended course to others; 80% strongly intended to use the information gained; 100% agreed that lectures (100%), readings (94%), activities (98%), pre-and post-test(94%), and face-to-face module (95%) helped their learning. 33% would like online interaction.	-
	Alexander L.K., 2008, USA, [25]	Pre-test: retrospectively in post-test; questionnaire with 5-point Likert-scale answer options. Post-test: questionnaire with 5-point-Likert-scale answer options	RR 177/156 (88) Self-assessed skills: - Use reports to identify health issues: 2.67 - Communicate with other agency to identify new cases: 3.04 - Maintain security and confidentiality of personal and PH information: 3.96 - Stay informed on PH laws and regulations: 2.77 - Use regulations to promote health: 2.69 - Recognize a disease outbreak: 2.79 - Collect biological or environmental samples: 2.30 - Be aware of amount of each important health problem: 2.76 - Work as part of a team during outbreak investigation: 2.77 - Write a press release: 2.06 - Create a line listing: 2.04 - Create an epidemic curve: 1.81	RR 177/156 (88) Self-assessed skills: - Use reports to identify health issues: 3.62 (p = < 0.0001) - Communicate with other agency to identify new cases: 3.87 (p = < 0.0001) - Maintain security and confidentiality of personal and PH information: 4.31 (*p* = < 0.0001) - Stay informed on PH laws and regulations: 3.63 (*p* = < 0.0001) - Use regulations to promote health: 3.55 (*p* = < 0.0001) - Recognize a disease outbreak: 3.89 (*p* = < 0.0001) - Collect biological or environmental samples: 3.17 (*p* = < 0.0001) - Be aware of amount of each important health problem: 3.49 (*p* = < 0.0001) - Work as part of a team during outbreak investigation: 3.89 (p = < 0.0001) - Write a press release: 3.28 (*p* = < 0.0001) - Create a line listing: 3.76 (*p* = < 0.0001) - Create an epidemic curve: 3.69 (*p* = < 0.0001) Satisfaction: 99% would recommend it; 96% thought the course was excellent; 100% inteded to use the information in their jobs; 100% said the material met the objectives; 98% thought that both internet, activities and case-studies helped their learning; 91% thought the face-to-face module helped. 4% thought the material did not fit together.	-
	Araz O.M., 2012, USA, [26]	Post-test survey with 5-point Likert-scale answer options and open ended questions	-	RR Unknown Satisfaction/ training methodology 66% found the multimedia presentations very helpful in terms of understanding the state of the outbreak; 69% agreed that the displayed scenarios were realistic and plausible; 84% agreed that the information exchange during the exercise was in high quality, which means that the decision-making environment facilitated communication.; “most particpants” agreed that the presented method increased readiness for a pandemic. “participants” stated that the video scenarios and the simulation model made it very real.	-
	Araz O.M. & Jehn M., 2013, USA, [27]	Pre- and post-test: 3-point-Likert scale answer options	RR 109/121(88) Self-reported capability: - Leadership and management: 93.5% - Mass care: 84.1% - Communication: 84.7% - Disease control and prevention: 91.5% - Surveillance: 82.0%	RR 109/121(88) Self-reported capability: - Leadership and management: 99.0% (*p* = 0.010) - Mass care: 85.6% - Communication: 94.1% (*p* = 0.001) - Disease control and prevention: 91.0% (p = 0.010) - Surveillance: 90.0% (*p* = 0.010)	.-
	Atack L., 2008, Canada, [28]	Pre- and post-test (< 2 weeks after): questionnaire with Likert-scale answer options and open-ended questions, adapted from validated questionnaire. Four validated questionnaires on organizational climate, receptivity, and course satisfaction: - Social System and the Organization (SSO) (climate) - New Ideas and You (NIY) (receptivity) - Education Program and Change (EPC) (feasibility) - Continuing Professional Education (CPE) (course satisfaction)	RR 67/76 (88) Knowledge test: Mean score of 38.4/56	RR 67/76 (88) Knowledge test: mean score of 46.4/56 (*p* = < 0.001) Knowledge & the system SSO: 75.6%; NIY: 85.6% EPC: 86.3% CPE:90.4% Relation between test and postcourse competency: SSO, NIY and CPE not sign. EPC: r = 0.431 *p* = 0.001. (80) Satisfaction: 100% agreed that the course was extremely helpful, 95% creative. 100% would recommend online learning as a way to learn about infection control; 27% thought video demonstration was in important to learning. Suggestions for approval: function to skip video’s (11%); not to re-do the entire module after failed for postquiz (7,5%). Open ended questions how the course had been useful: three major themes: improved hand hygiene practice, improved teaching to patients/visitors/staff on how to use PPE, improved their own techniques RR 55/76 (72) Behavioral change: reported an example of behavioral change after the course.	-
	Atlas R.M., 2005, USA, [29]	Scored evaluation and anecdotal comments	-	RR unknown Participants regularly identify the moulaged patients as the most effective element of training. Self-assessed attitude: 94% say use of patient simulator changed their awareness of respiratory disease transmission, Self-assessed Behavior: 98% would alter respiratory protection when confronted with a patient demonstrating signs and symptoms of a respiratory tract infection.	-
	Baldwin K., 2005, USA, [30]	Post-test: 5–10 MCP knowledge questions after each module. + unknown qualitative evaluation method for satisfaction	-	RR 15/15(100) Knowledge Results not reported Satisfaction/training methodology: Challenges: Overly complex system of accessing different parts of the program; impossible to predict large number of updates required; internal shift of focus could not be implemented; funding to sustain the program was hard; lack of designated ownership of the program. Benefits: Use of an readily available system, accessible at different sites; completed at own pace and role; evaluation of compliance could be monitored; individuals could be identified for additional training.	-
CBS	Bazeyo K.M., 2015, Uganda, [31]	Pre- and post- knowledge test. Format unknown.	RR 330/unknown Knowledge: Mean score 5–20% lower than in post-test for all districts. No numerical results available.	RR 330/unknown Knowledge: Mean score 5–20% higher than in the pre-test for all districts. No statistical results available.	–
	Becker K.M., 2015, Ghana, Uganda, Nigeria, USA, [32]	Post-test: summing up achievements	-	RR 43/43(100), two years. System performance: Built in-country cadres of midlevel public health; cross-border rabies vaccination program; evaluation of 66 disease surveillance systems; integrated vaccination campaigns & improved coverage rates; increased capacity for epidemiological studies, provide an evidentiary basis for PH action/strategy; influence PH policy; improved diagnostic and laboratory management skills; participants trained district level laboratory personnel in integrated disease surveillance.	-
TOT	Berrian A.M., 2018, USA, [33]	Pre- and post-test: Written MCP knowledge test on content; Post-test: written self-report (4-point Likert-scale) of knowledge, skills, pedagogical skills, leadership skills Follow up: -	RR 10/10(100) Knowledge: No data Self-assessed knowledge: No data Self-assessed skills: No data	RR 10/10(100) Knowledge: Mean increase of 17% p = 0.0078; Self-assessed knowledge: Improved on pathogen transmission (*p* = 0.0156), risk mitigation (p = 0.0020) and effective pedagogy (*p* = 0.0020). Self-assessed skills: Improved for risk assessment *p* = 0.0078; facilitate a workshop (p = 0.0020).	Results from the second wave were part of the train-the-trainer session, and are not presented because they were non-professionals.
	Biddinger P.D., 2010, USA, [34]	Pre- and post test of knowledge and confidence; Post-exercise survey to assess awareness of agencies’ roles and responsibilities; AAR	RR 1145/5892 (participants of table-top exercises) Knowledge: no data available; Attitude: no data available.	RR 1145/5892 (participants of table-top exercises) Knowledge: increased with 25% on average. 56% increase knowledge of what other agencies may have to offer (resources/assets); 69% clarify role&responsbility of perosn & organizaiton; Attitude: On average 12% better confidence. Satisfaction/ training methodology:77% scored it effective in practicing working together; 73% providing an opportunity to evaluate plans/procedures; Satisfaction for target groups: Regional response exercise respondents reported higher satisfaction with the effectiveness of understanding roles (*p* < 0.001), providing right environment (*p* < 0.001), promoting regionwide cooperation and mutual aid (*p* < 0.001); more engaged in the exercise (*p* < 0.006). No relation in knowledge and satisfaction with the program. Small and big towns had higher satisfaction. Network building: 70% assessing connectivity within/across agencies; 53% promoting region-wide cooperation and mutual aid.	-
	Cathcart L.A., 2018, USA, [35]	Pre-, post- and follow-up (timing unknown): survey with 5–8 statements rated with a 5-point-Likert-scale answer options.	120/120 (100), Self-assessed skills Mean score 2.2	120/120 (100), Self-assessed skills Mean score 4.0	59/120 (49) Self-assessed skills Mean score 4.3 Self-assessed behavior: Used what I learned: mean score 4.2 +/−1.0 Different way of conducting my job: 3.9 +/− 1.0; Satisfaction: Prepared me for my job: mean score 4.0 +/− 1.0
	Chandler T., 2008, USA, [36]	Knowledge test of 15 questions (unknown methodology) Self-assessed knowledge on a 4-point-Likert-scale Skills and behavior assessed by local supervisors (unknown methodology)	817/> 817 Knowledge: mean score 72.17 (SD 16.31)	RR 817/> 817 Knowledge: Mean score 94.25 (SD 8.07) (*p* < 0.001). RR 764/> 817 Self-assessed knowledge: 86% felt more knowledgeable about the basic emergency preparedness core competencies; 82% about their agency’s chain of command during an emergency response; 80% about their own functional roles during emergency reponse. RR 511/> 817 Skills No data available RR 511/> 817 Behavior Supervisors: usage of the course was an effective means for improving work performance.	-
	Chiu M., 2011, USA, [37]	Survey in which self-assessed confidence in preparedness, response and recovery skills and needs were rated using a 5-point-Likert-scale	41–54/182 (23–30) Self-assessed skills: 10 preparedness competences: 30.2 SD6.7 8 response competences: 26.0 SD5.5 7 recovery competences: 23.1 SD5.5 Training needs Preparedness: 32.7 SD7.3 Response: 26.0 SD6.9 Recovery: 22.6 SD6.5	41–54/182 (23–30) Self-assessed skills: 10 preparedness competences: 36.2 SD4.9 (*p* < 0.001) 8 response competences: 30.9 SD4.5 (*p* < 0.001) 7 recovery competences: 28.8 SD4.18 (*p* < 0.001) Training needs Preparedness: 22.0 SD9.5 (*p* < 0.001) Response: 17.1 SD7.3 (*p* < 0.001) Recovery: 15.7 SD6.6 (*p* < 0.001)	-
	Craig A.T., 2007, Australia, [38]	Post-test: feedback from stakeholders in debriefing sessions; state-wide participant questionnaire with 5-point-Likert-scale answer options; both retrospective pre-test and post-test.	RR unknown Self-assessed system performance: Prepared to respond to a case of pandemic influenza: mean score 3; > 55% agreed or strongly agreed.	RR unknown Self-assessed system performance: Prepared to respond to a case of pandemic influenza: mean score 4; > 90% agreed or strongly agreed Satisfaction: 84% agreed that exercise Paton prompted their influenza pandemic planning.	-
	Dausey D.J., 2007, USA, [39]	Review of after action reports, participants post-exercise evaluations, and internal team discussions and consensus following exercise debriefings	-	RR 513/unknown Exercise methodology: Exercises should a) be designed to achieve a specific objective. B) be as realistic as possible & logistically feasible; C) be designed around “issue areas” rather than scenarios. E) have forced, targeted and time delineated decision making. F) have limited number of participants. G) be designed and executed from collaborative engagement of representatives from participating agencies and external developers and facilitators.	-
CBS	Dausey D.J., 2014, USA, [40]	Post-test: evaluation form with Likert-scale answer options, some with open-ended questions Follow-up: semi-structured interviews with health leaders at ministry level	-	Unknown/558 participants; Satisfaction/methodology: 88–100% rating the quality of the exercise as high; 92–94% exercise helps to understand roles and responsibilities of organizations responding to influenza pandemic; 50–73% ability to identify key gaps in performance.; more sectors should be included, including more private partners and NGOs; better grounding of theoretical responses with practical responses are required. Self-assessed knowledge: 82–100% gain knowledge that they plan to improve the preparedness of their organization; Self-assessed attitude: the exercise raised awareness and understanding about public health threats;	unknown 137 observers Attitude Participation in the exercise helped to motivate to develop an exercise program and regularly assess different aspects of their public health preparedness. Barriers are a lack of financial resources, limited support among leadership to develop and sustain the program. Behavior: Most countries reported modifying and using exercise templates.
	Dickmann P., 2016, Hong Kong, Poland, Sweden, Switzerland, UK, [41]	Pre-and post-questionnaires; day assessments at the end of each day	RR 15/15	RR 15/15 Satisfaction: 14/15 expectations have been fully met; they appreciated that the training was based on a reflective and reframing approach Self-assessed knowledge: 14/16 stated their understanding had increased considerably	-
			Self-assessed knowledge: 50% good knowledge of risk communication theory.	Self-assessed attitude: the part that had good knowledge, felt better prepared to advocate for this change	
CBS	El-Bahnasawy M.M., 2014, Egypt, [42]	Pre- and post- knowledge tests (methodology and scales unknown)	RR 125/unknown Knowledge: median scores: Anthrax 3a; Tick-borne relapsing fever: 2a; Lice-borne relapsing fever: 2a; *Clonorchis sinensis*:4a	RR 125/unknown Knowledge: median scores: Anthrax 8b; Tick-borne relapsing fever: 4b; Lice-borne relapsing fever: 4b; Clonorchis sinensis: 9b Satisfaction/methodology: results useless because no information about training methods is provided.	RR 125/unknown Knowledge: median scores: Anthrax 9c; Tick-borne relapsing fever: 5c; Lice-borne relapsing fever: 5c; Clonorchis sinensis: 9b (p < 0.001 for all subjects)
TOT	Faass J., 2013, USA, [43]	Post-test: Partly hardcopy, partly online survey with 29 open-ended and 5-point-Likert-scale questions	–	RR 120/231(52): Satisfaction: 90% of attendees rated the overall impression as “good” or higher. 94% answered “agree”/ “strongly agree” on relevance of course materials to their organizations and employees. Attitude: 84% agreed that pandemic preparedness posed a challenge for the transit industry;	Results from the second wave of training: -
	Fowkes V., 2007, [44]	Post-test evaluation form with 5-point-Lickert-scale answer options and open-ended question: How would you incorporate what you have learned?	-	RR > 6000/9537 Satisfaction/methodology: 95% rated modules as good/excellent Self-assessed attitudes: modules reinforced needs for emergency plans; enhanced awareness of possible unusual clinical presentations; enhanced consciousness about infection control. Self-assessed knowledge: modules provided useful approaches to decontamination;	-
	Fowkes V., 2010, [45]	Evaluation forms; assessment of the departments’ emergency plans; AARs completed by the exercise groups; debriefing of coordinators; interviews with trainees; follow-up interviews; quarterly reports by the organizing party	RR 1176/1496 System results: 37% of emergency operation plans complete	RR 1176/1496 System results: 46% of emergency operation plans complete Satisfaction/methodology: 92–98% rating the training experiences as good to excellent.; Self-assessed knowledge/skills: 92–98% rates knowledge and skills gained form the exercises as good to excellent.; Skills: 72–90% were able to describe events and steps necessary to activate their plan, actions that should be taken when it is activated, roles of individuals, internal and external communications needed, how to participate in a coordinated response, and who is responsible for the oversight of the plan. 69% how to correct the plan when needed; 62% to lessen the spread of disease to staff, patients, and families. 42% to plan for a surge of infectious patients.	RR 1176/1496. System results: 74% of emergency operation plans complete; 91% of clinics made improvements in their emergency plans compared to post-test with statistically significant changes in 2/3 of the 15 criteria (p = 0.001–0.46).
	Gershon R.R., 2010, [46]	Pre- and post-test of 7 questions on knowledge and 9 items course evaluation.	RR 129/unknown Knowledge: mean 6.3 +/− 1.1	RR 129/unknown Knowledge: mean 6.6 +/−0.8 (*p* < 0.001) Satisfaction: 98% thought the program was valuable; 97% risk was addressed; 95% length of training is acceptable. Self-assessed knowledge: 95% their PPE understanding was reinforced; > 92% knowledge of respiratory illnesses improved;	-
TOT	Grillo M., 2017, [47]	1 h pre- and post test with 40 MPC and true/false questions testing knowledge.	136/136 (100) Knowledge: 50.5% mean score	136/136 (100) Knowledge: 67.9% mean score (p < 0.001)	Results from the second wave of training: -
	Hegle J., 2011, [48]	Observation by at least 2 researchers using an observation guide; semi-structured interviews with exercise leaders; review of planning and exercise materials		Unknown/682 System performance: Direct result of the exercise: 1) building relationships among response partners across counties and agencies; enhancing social capital. 2) promoting visibility of public health and assets in an emergency response; 3) testing multiple communication systems. The use of these systems was problematic in all exercises observed; 4) training public health practitioners in exercise evaluation.	-
	Hoeppner M.M., 2010, USA, [49]	At 6 and 12 months after participation in one of the courses, a survey with eight questions. Skills/knowledge gained, changed attitudes, changed behavior, affected others, face barriers. Based on the Kirkpatrick model	-	-	RR 244/387 (63) Satisfaction: 6- months: 64% agreed that courses helped them develop the competencies; 12 months: 63.5%. Self-assessed knowledge/skills: Development of critical thinking and systems-thinking. Deeper appreciation for the complexity of planning and response and the need for collaboration across all agencies and levels of government. Recognised the national incident management system as a organizing mechanism. Better able to gauge the resources needed and better advocate for these. Better able data collection; better able to inform and educate others. Self-assessed attitude: Recognized the need for improved surveillance, use of technology to organize data, shared data management across agencies. Behavioral change: Performed better data collection; inform and educate others; developed wider range of exercises, revised emergency plans, enhanced and eexpanded cross-agency collaboration. Barriers: financial and human resources, lack of time, unavailable software, how to convince others?, underutilization of expertise due to changing jobs.
	Horney J.A., 2005, USA, [50]	Knowledge post-test after each module; survey;	-	416/3030 (14) Knowledge: no results available Satisfaction: 98% indicated that the training module provided the information they were looking for (100% for PH nurses); 62% had recommended the course to collegues. Self-assessed knowledge: 2/3 module introduced them to terms and concept they were previously unfamiliar with; 87% module clarified terms or concepts they had not enough information about; 97% module reinforced familiar terms and concepts; 81% module specifically adressed prof roles and responsibilities (80% for PH nurses); Self-assessed attitudes: 92% module made them feel better equipped to do their job (94% for PH nurses);	-
	Hueston W.D., 2008, USA, [51]	Representatives of partner universities shared their lessons learned.	-	-	Unknown Several years after the start of the program: Methodology: Strong collaborations and good communication are essential; differences in DVM and MPH professional cultures must be understood; differences in CVM and SPH organizational cultures must be bridged; human and financial resources provide significant challenges; faculty efforts systems may differ; different curriculum design and delivery call for innovative approaches; CVM/SPH partnerships are mutually beneficial; Technology is not a panacea - developing good distance learning courses takes a lot of time and money; no single standard for distance learning exists to unify the competing software platforms and various computer operating systems.; be prepared for succes.
	Johnson Y.J., 2009, USA, [52]	Post-exercise evaluation form with 11 five-point-Likert-scale questions	-	RR Unknown Satisfaction/ Training methodology: Exercise was well structured and organized (4.0); scenario was plausible & realistic (4.14); knowledgeable facilitators about (4.14); facilitators kept exercise on target (3.86); technologies were useful (4.14); participation was appropriate for my position (4.00); correct mix of people (3.79); adequate facilities utilized (4.36); meals and breaks (4.71); Self-reported skill: could practice and improve responsiveness (3.86); after the exercise my agency/jurisdiction is better prepared to successfully deal with the scenario (4.00).	-
	Kohn S., 2010, USA, [53]	Observation. Post-test: evaluation sheet with Likert-scale answer options; informal conversations with participants and trainers.	-	RR 213/unknown 80% agreed enough interactive exercises; 89% that session provided useful information now/ in near future; 90% that overall session was valuable.; conversations identified: participants saw the course as effective and helpful in their professional setting; overwhelming volume of information; wish for more examples/ scenarios to illustrate practical application. ‘Many’ thought the material as not relevant to them. INFLUENZA TRAINING: RR unknown/37 Positive ratings of course content, facilitator, and presentation; information provided helped frame their emergency response roles & responsibilities within a PH context; training is essential in clarifying some response activities; core concepts taught help operate more effectively during a variety of future emergencies; still the course material could have been better tailored.	-
TOT	Livet M. 2005, USA, [54]	Participants surveys using 6-point-likert scales; observation forms; and evaluation instrument for a TTX	67–70/80 (84–88) Networking/relationship building: 1.60 – 4.10 on a 6-point scale Competence: Describe PH role during emergency response: 4.09 Describe chain of command: 4.58 Identify response plan: 5.06 Describe functional roles: 5.07 Describe communication roles: 4.61 Identify limits to own competence: 4.48 Recognize unusual events: 4.66 Apply creative problem solving and evaluate effectiveness: 4.62 Plan a TTX: 3.84 Implement a TTX: 3.80 Evaluate a TTX: 3.76 Write an AAR: 3.72 Describe the ICS: 4.62 Describe risk communication strategies: 4.04 Describe criminal and epidemiological investigative methods: 3.84	RR 67–70/80(84–88) Satisfaction: Organization of the sessions and TTX 60–90% thought it good-excellent. Usefulness and applicability 60–84% agreed or strongly agreed. Networking/relationship building: 4.91–5.67 on a 6-point scale (*p* < 0.001) Competence: Describe PH role during emergency response: 5.30 (*p* < 0.001) Describe chain of command: 5.35 (p < 0.001) Identify response plan: 5.57 (*p* = 0.001) Describe functional roles: 5.49 (p < 0.001) Describe communication roles: 5.31(p < 0.001) Identify limits to own competence: 5.30 Recognize unusual events: 5.40 (p < 0.001) Apply creative problem solving and evaluate effectiveness: 5.26 (p < 0.001) Plan a TTX: 5.44 (p < 0.001) Implement a TTX: 5.42 (p < 0.001) Evaluate a TTX: 5.36 (p < 0.001) Write an AAR: 5.23 (p < 0.001) Describe the ICS: 5.46 (p < 0.001) Describe risk communication strategies: 5.29 (p < 0.001) Describe criminal and epidemiological investigative methods: 5.20 (p < 0.001)	Results of the second wave of training: -
	Macario E., 2007, USA, [55]	Broadcast evaluation through a survey and knowledge post-test for those who wished further education; Tabletop evaluation through participant & facilitator surveys; telecall interview	-	RR: broadcast 821/25000 RR: tabletop 164/unknown RR knowledge test: 735/25000 RR teleconference: 21/unknown Satisfaction: 75–98% agree on satisfaction statements for the broadcast; 4.1/5 tabletop is helpful in feeling more prepared; 4.1 for tabletop overall. 2 h was too short for real effectiveness. Tabletop helped to learn the language of such preparedness activities and identified major gaps; 4.1 on how deliverables helped them in familiarizing themselves with pp. plans; 3.7 for broadcast info; Survey tabletop facilitators: 4.7 program was appropriate for their health departments; 4.3 training helped increasing capacity to respond; program helped to identify key deficiencies in preparedness, as well as to learn what is and is not in their control. Future tabletops address more localized concerns and have materials earlier available; Knowledge: 90% had 100% score; survey tabletop particpant: teleconference satisfaction, skill, confidence: pairing of broadcast with tabletop was effective complementary training; training helped them better understand their plans, roles of decion makers in the chain of command;	-
CBS	Martin G., 2018, Ireland, [56]	Lessons learned in hot debrief + observers/evaluators during the exercise		Unknown/> 200 Methodology: Exercise planning should not be overly ambitious. If testing too many facets of the emergency response protocols, the public health response can be deprioritised; the practical implementation of communication protocols in a real time exercise of this scope proved challenging; the roles and responsibilities of the various agencies involved needs to be clear. In the chaos of an incident it is easy for role confusion to set in; equipment and infrastructure must be in place and must have been thought about before an actual incident (wheter ore not cell phone signals are available on site or requiring boosting e.g.).	
	Mitka M., 2003, USA, [57]	Comments of participants	-	Reaction: exercise helped to improve the nation’s response by testing emergency plans and finetune them.	-
	Morris J.G., 2012, USA, [58]	Post-test: survey 6 open-ended & 10 questions with Likert-scale answer options	-	RR 22/unknown Satisfaction/methodology 22/22 scenario plausible, 20/21 scenario comprehensive, 22/22 scenario generated productive discussion, 22/22 scenario helped identify strength/weaknesses, 17/22 scenario helped identify gaps in current planning, 19/21 helped build relationships with participant from key agencies, 16/20 relationships with participants from other states Knowledge 22/22 knowledge of this type of emergency event has increased, 17/22 understanding of my role has increased, 22/22 my understanding of others’ role has increased.	-
	Olson D., 2008, USA, [59]	Post-test: Course evaluations (no results) Follow-up: 6 & 12 months after end of program (no results) Testimonials (unknown method) Based on the Kirkpatrick Model	-	RR unknown/1680 Satisfaction: “I would not be in the position I am today if I had not had the opportunity to advance my knowledge in their uniquely structured program” “It has helped me in everything I do related to emergency preparedness!” Behavior: “I have reviewed and updated our plans. I have trained staff on the topics I studied. We have addressed mental health needs more in our planning and training.” Self-assessed knowledge: These courses have made me understand what other training I need as well as training needed by other staff and partners in preparedness. “Gave me more knowledge about writing plans, designing plan exercises, working with media and developing media kit, discussing mental health issues with local providers, planning for emergencies with community leaders.	-
TOT	Orfaly R.A., Frances J.C., 2005, USA, [60]	Evaluation of trainings performed by the TRAINED-TRAINERs 6 months after the TTT: Survey; Open-ended questions; registered number of trainings and people trained in second wave; follow-up interviews with TRAINED-TRAINERs	-	-	TRAINED-TRAINERs RR: 21/21 (100) Participants RR unknown Behavior: 118 participants trained; 20% of TRAINED-TRAINERs had conducted a training themselves after 6 months. Barriers were: lack of time&resources; not enough confidence; trainings were too general and did not provide enough information specific to particpants’ funcitonal roles Satisfaction: high rankings for the instructors (4.5/5); similar high scores for knowledge, responsiveness and organization; content and materials 4.0; self reported findings on training objectives:> 4.0 for four of the trainers and 3.5 for the fifth; qualitative evaluation of participants were favorable;
	Orfaly R.A., Biddinger P.D., 2005a, USA, [61]	Evaluation survey (unknown method)	-	24/24 (100) Satisfaction: 96% would recommend the course to classmates; 92% the course is superior to other courses they had taken at HSPH; 100% the course is useful to their profession; 83% there was appropriate emphasis on practical skills.	-
TOT	Otto J.L., 2010, USA, [62]	Post-test: survey with MCP/Lickert scale questions; Follow up in six months with a survey with MCP and open questions on implemented trainings, revised plans, and barriers	-	RR 65/85 (76) Satisfaction/methodology: 95% expectations & instructions clearly presented, 92% scenario is realistic and credible, 87% TTX better equiped them to plan and execute an installation-level pandemic influenza exercise. 89% TTX identified strengths and gaps in response, 77% identified consequences of interventions and described strategies for dealing with them. 94% TTX identified opportunities for enhanced military and civilian coordination.	50/85 (59) Behavior: 68% had a new/revised pandemic influenza plan, 44% my installation conducted a pandemic influenza exercise. Barriers: 67% competing priorities, 37% time limitations, 19% personnel limitations, 11% budget limitations, 37% other.
	Peddecord K.M., 2007, USA, [63]	Pre-post- and 6-week follow-up: questionnaire with MCP, likert-scale and open-ended questions. Pre-and post on knowledge Follow-up: attitudes + behavior	520/> 1658 (< 31) Knowledge: mean score 81% Attitudes: 76% thinks mass vaccination clinics are necessary and beneficial 8% thinks it is a good idea but not that important in the overall PH system 10% feels intimidated by the scope& responsibilities 3% ambivalent 3% no opinion	520/> 1658 (< 31) Knowledge: mean score 85% (*p* < 0.001) Attitudes 92% thinks mass vaccination clinics are necessary and beneficial 3% thinks it is a good idea but not that important in the overall PH system 4% feels intimidated by the scope& responsibilities < 1% ambivalent < 1% no opinion (p < 0,001 compared with pre-test)	291/> 1658 (< 18) Attitudes: 84% thinks mass vaccination clinics are necessary and beneficial 4% thinks it is a good idea but not that important in the overall PH system 8% feels intimidated by the scope& responsibilities 2% ambivalent 2% no opinion (*p* = 0.002 compared to pre-test, *p* = 0.005 compared with post-test) Behavior: 44% planned or taken action to change their health departments’ emergency preparedness plan
	Potter M.A., 2005, USA, [64]	Post-test: survey with Likert-scale answer options, results of own project		28/28(100) Satisfaction/methodology: high scores, see study in Table 1. Knowledge & skills: Mean scores between 4.00 and 4.93 for assessing change potential; system thinking; improving negotiation skills; solution mapping; legal mandates; implication wheel, interest-based dispute resolution.	-
	Quiram B.J., 2005, USA, [65]	Pre- and posttest: knowledge test;	Unknown/167 Knowledge: see data post-test	Unknown/167 Knowledge: increases on all topics. For different sessions: overall: between + 9.7 and + 32.2% Preparedness planning: between + 5.8 and + 24,5% Biological/chemical agents: between + 3.9 and 34.3% Laboratory capacity: − 2.4 and + 49.5% Risk communication: + 4.6 and 30.2% Communication and IT: + 12.9 and 47.1% Surveillance & epidemiology:+ 12.7 and + 45.6%	-
	Qureshi K.A., 2004, USA, [66]	Pre-post- and follow-up (1–6 months after) had 10 MCP questions on knowledge, 8 on behavioral intentions and attitudes using 3-point-likert scales General evaluation to a random sample of 100 people	678/764 (89) Knowledge: means score 8.24 Attitude: 84.3% important to respond during emergency 91.0% responding will help the community 72.9% other PH nurses will respond during an emergency 78.9% they are themselves responsible to assist during emergency 73.2% intend to respond when needed 70.1% coworkers approve my role in emergency response 59.2% believes significant other approves role in emergency response	726/764 (95) Knowledge: mean score 8.38 (*p* < 0.05) Attitude: 85.3% important to respond during emergency (NS) 91.6% responding will help the community (NS) 76.9% other PH nurses will respond during an emergency (*p* < 0.05) 84.6% they are themselves responsible to assist during emergency (*p* < 0.01) 76.5% intends to respond when needed (p < 0.05) 73.0% coworkers approve my role in emergency response (NS) 64.1% believes significant other approves role in emergency response (p < 0.05) RR 94/100 (94) Satisfaction: 92% program was clear; 88% well-organized, 72% reinforced knowledge regarding emergency response	230/764 (30) Knowledge mean score unknown Attitude: 93.5% important to respond during emergency (p < 0.05 with post) 94.0% responding will help the community (NS with post) 83.6% other PH nurses will respond during an emergency (NS with post) 91.8% they are themselves responsible to assist during emergency (NS with post) 26.2% intends to respond when needed (NS with post) 81.5% coworkers approve my role in emergency response (NS with post) 73.4% believes significant other approves role in emergency response (p < 0.05 with post)
	Rega P.P., 2013, USA, [67]	Pre- and post-exercise survey with 5-point-Likert-scale answer options about knowledge, satisfaction	Self-assessed knowledge: few participants reported ‘excellent’ knowledge	RR unknown Self-assessed knowledge: 96–100% improved knowledge Satisfaction: ‘most participants’ valued the exercises for their usefulness and content; exercises are innovative, entertaining, educational; was recommended to be delivered to fellows.	-
CBS	Richter J., 2005, USA, [68]	Post-test: survey collecting qualitative and quantitative data	-	32/50(64) Satisfaction/methodology: majority like small groups; all agreed the workshop would benefit their job. Majority information was useful and met expectations. Self-assessed knowledge: All agreed increased understanding of PH response protocols during outbreaks. 91% agreed better understood protocol during BT attack. Majority had gained greater understanding in public safety, law enforcement and security response protocols (97%) and a BT attack (94). 100% learned about others’ roles during outbreak, 96% during BT attack. Networking: 95% workshop enabled make contacts and network with staff with similar positions at different agencies. 97% had opportunity to network with different positions.	–
	Rottman S.J., 2005, USA, [69]	Pre- and post-test: survey	RR 403/unknown Knowledge: 75,5% mean test score Self-reported knowledge: no data available	RR 403/unknown 87.4% mean test score (*p* < 0.001). Known own and health department’s roles in disaster: increased (*p* < 0.001).	-
	Sandstrom B.E., 2014, Sweden, [70]	Observation of the exercises; evaluation seminars after the exercise	-	Satisfaction/Methodology: applicability of the model was independent of the type of scenario, i.e. it could be used as a generic tool for exercises.; First test (3p): Mutual recognition of the scenario site benefitted participants; Discussion concerning preparedness took 1/3 of the exercise.; important having a highly professional exercise director; high flexibility of the concept; possibility to concentrate the exercise on a few cards without losing the strength of the exercise; participants considered it a cost-effective way of performing table-top exercises; suggested time-frames were removed. Second test (> 40 p): used as a tool to raise awareness; in a heterogeneous population overall understanding of different organizations needs and limitations is possible; not efficient for going into deeper detail in specific procedures and plans.; With a large audience, merely spectators, ineffective for experienced personnel; high flexibility of the concept supported conducting a multi-agency exercise on a more overarching level; allowed for giving different timeframes for various categories of participants; a few more topics were included.; Third test (35p in groups 5–7): the scenario was easily adapted to serve in an international emergency response exercise.; different phases of the response could be emphasized in depth by applying the Director card issues and the corresponding topics.; the tool supported emphasis on different areas of operations in specific phases.; participants confirmed the tests before and appreciated the simplicity of the exercise card concept; also usable for international cross-border exercises.	-
	Sarpy S.A., 2005, USA, [71]	Pre-and post-test: survey with Likert-scale answer options on behavior, attitude) Post-test: additional Likert-scale questions on self-assessed objectives, satisfaction and open questions on most valuable and useable parts	49/49 (100) Self-assessed skill Prepared to effectively respond to a SARS event: 3.98(1.52) Community is prepared: 3.10(1.39) Recognize a SARS outbreak: 4.53(1.40) Establish contact and coordinate others: 5.02(1.18) Maintain effective protocols for roles& responsibilities: 4.65(1.33) Use the chain of command: 5.24(1.22) Communicate relevant information in/externally: 5.37(1.20) Determine communication to public: 4.76(1.51) Determine communication to media: 4.63(1.51) Monitor progress and action: 4.71(1.30) Use investigation& management strategies: 4.88(1.31) Self-assessed attitude SARS is threat to my community: 5.65(1.15)	44/49 (90) Self-assessed skill Prepared to effectively respond to a SARS event: 5.20(1.13) p < 0.001 Community is prepared: 3.93(1.26) NS Recognize a SARS outbreak: 5.75(0.92) p < 0.001 Establish contact and coordinate others: 5.73(0.90) p < 0.001 Maintain effective protocols for roles& responsibilities: 5.32(1.05) NS Use the chain of command: 5.80(0.93) p = 0.002 Communicate relevant information in/externally: 5.86(0.88) NS Determine communication to public: 5.41(1.15) NS Determine communication to media: 5.45(1.13) *p* = 0.004 Monitor progress and action: 5.41(1.00) p = 0.003 Use investigation& management strategies: 5.36(0.99) NS Self-assessed attitude SARS is threat to my community: 5.82(1.02) NS Self-assessed knowledge: improved understanding of biological agents: 5.34/7 Improved understanding of functional roles & resp.: 5.57/7 Satisfaction (selection): Content: 5.88; format 5.77; mix of participant:5.86; pre-ttx lecture:6.36; ttx: 5.93; overall effectiveness: 6.12 Qualitative answers: most valuable: mix of participants, experts present, discussion sessions, networking possibility. Relevance to job because of: symptom identification, communication, in-& external partners. Improvements on: individual answers, missing perspectives, missing formalized introductions, packaging for greater dissemination.	-
	Savoia E., 2009, USA, [72]	Pre- and post-test surveys with Likert-scale answer options and % of substantive answers	56/89(63) Knowledge Between 25 and 85% substantive answers for different questions Confidence See results post-test	56/89(63) Knowledge Between 70 and 100% substantive answers for different questions (*p* < 0.05 in all topic areas) Confidence Legal authorities: Between the 32 and 46% difference between pre-and post-test (*p* < 0.05 for all topic areas) Policies and procedures: Between 30 and 43% difference between pre- and post-test (p < 0.05 for all but one topic (declaration of emergencies).	-
	Savoia E., 2013, USA, [73]	Guided group discussions where lists of recommendations were developed	-	RR unknown Training methodology: Include practitioners playing key leadership roles in the real world; representatives from agencies and disciplines across the range of jurisdictions that would respond to a specific public health threat; senior level players and specific agencies; a plausible scenario and timeline; clear an measurable exercise objectives; link exercises to prior years’ efforts and prior-tested capabilities; Expertise in exercise planning is a limited or unavailable resource in local health departments; heterogeneity in scopes has implications for the exercise design, required level of participation, and the approach to evaluation.	-
TOT	Soeters H.M., 2018, Guinea & USA, [74]	Pre-and post-test with 30 MCP questions on knowledge. Post-test demonstration of skills	1625/1625(100) Knowledge: Median score 17	1625/1625(100) Knowledge: median score 25 Skills: donning/doffing: 70% ‘acceptable’ score for HCW, 97% for supervisors, 83% for trainers Chlorinated water preparation: 80% for HCW, 79% for supervisors, 81% for trainers.	
	Taylor J.L., 2005, USA, [75]	Comments of participants during exercise; post-test: written evaluations (unknown methods)	-	69/150 (46) Self-assesed attitude: particpants realized needs to continue to build surge capacity specific to the challenges of an influenza pandemic; elected officials and decision makers must have clear understanding of the potential implications of an influenza pandemic and the additional efforts are needed to assure that such officials are adequately informed; pandemic influenza planning needs to be further coordinated with the existing emergency response infrastructure and additional training in incident command is needed; More detailed operational planning is required to achieve an effective overall response; additional support is needed at the federal level	-
	Umble K.E., 2000, USA, [76]	Pre- and post-tests and follow-up (3 months): Knowledge 5 MCP questions; Agreement, Self-efficacy, Adherence, Setting factor, awareness using Likert-scales. The model used to examine the program’s effects was rooted in several health behavior theories, including the health belief model,15,16 social cognitive theory,16–18 the transtheoretical model,19,20 and the theory of reasoned action.	RR 196/470 for classroom Knowledge: Polio schedule 3.82/5 (1.40) Attitude: Agreement with the polio schedule 33.80/40 (5.55) Adherence to general recommendations: 11.50/15 (4.22) Adherence to the polio schedule 9.30 (5.71) Self-assessed skill: self-efficacy of the polio schedule 4.68/8 (2.09) RR 116/251 for broadcast Knowledge: Polio schedule 2.68/5 (1.61) Attitude: Agreement with the polio schedule 32.37/40 (5.91) Adherence to general recommendations: 9.50/15 (4.49) Adherence to the polio schedule 11.84 (4.31) Self-assessed skill: self-efficacy of the polio schedule 3.53/8 (1.99)	RR 196/470 for classroom Knowledge: Polio schedule 4.48/5 (0.89) (*p* < 0.001) Attitude: Agreement with the polio schedule 38.38/40 (2.27) (*p* < 0.001) Adherence to general recommendations: unknown Adherence to the polio schedule: unknown Self-assessed skill: self-efficacy of the polio schedule 6.93/8 (1.84) (p < 0.001) RR 116/251 for broadcast Knowledge: Polio schedule 4.19/5 (1.16) (p < 0.001) Attitude: Agreement with the polio schedule 37.37/40 (3.33) (*p* < 0.001) Adherence to general recommendations: unknown Adherence to the polio schedule: unknown Self-assessed skill: self-efficacy of the polio schedule 6.55/8 (1.96) (p < 0.001) Significant (p = 0.006) higher increase in knowledge for classroom education compared to broadcasts.	RR 196/470 for classroom Knowledge: Polio schedule 4.52/5 (0.85) (p < 0.001 with pre) Attitude: Agreement with the polio schedule 37.36/40 (2.71) (p < 0.001 with pre) Adherence to general recommendations: 12.38/15 (3.36) (p < 0.05 with pre) Adherence to the polio schedule 16.25 (4.90) (p < 0.001 with pre) Self-assessed skill: self-efficacy of the polio schedule 6.88/8 (1.64) (p < 0.001 with pre) RR 116/251 for broadcast Knowledge: Polio schedule 4.24/5 (1.07) (*p* < 0.001 with pre) Attitude: Agreement with the polio schedule 37.01/40 (3.60) (p < 0.001 with pre) Adherence to general recommendations: 11.84/15 (4.31) (*p* < 0.05 with pre) Adherence to the polio schedule 14.42 (5.44) (*p* = 0.084 with pre) Self-assessed skill: self-efficacy of the polio schedule 6.55/8 (1.96) (p = 0.001 with pre) No significant difference between classroom and broadcast
	Waltz E.C., 2010, USA, [77]	ARS: post-test evaluation form Broadcasts: # views Web-based lecture: post-test evaluation form	-	ARS RR 93/93 (100) Satellite broadcast RR none Web-based education RR 20.000/44.000 (48) Satisfaction: ARS: 95% agreed that the technology was beneficial to the training; Broadcast: 3871 views on average where in three years for some courses the number of later views exceeded the live views.; Web-based education: 96% rated course quality as good, 99% would recommend the course, 88% the course would help perform their job more effectively	-
	Wang C., Wei S., Xiang H., Wu J., 2008, China, [78]	Pre-, post- and follow-up (12 months) tests of 40 MCP questions (knowledge) and 5-point-Likert scales for skills. Observation by colleagues, and evaluation of a subsequent real outbreak.	RR 41/43 (95) Knowledge: mean score 21.62 +/− 6.37 Self-assessed skills: mean scores between 2.54–3.05	RR 41/43 (95) Knowledge: mean score 31.59 +/− 5.85 (p < 0.01 with pre-test) Self-assessed skills: mean scores between 3.49–4.12. Increase in mean scores for all individual skills (*p* < 0.01) Satisfaction/ Training methodology: 90% thought the training to be excellent. 98% very satisfied with venue, logistics, communication.	RR 41/43 (95) Knowledge: mean score 32.39 +/− 5.15 (p < 0.01 with pre-test) Increase *p* < 0.05 for assessment knowledge post vs. follow-up test Self-assessed skills: mean scores between 3.68–4.07. Increase in mean scores for all individual skills (p < 0.01) compared with pre-test
	Wang C., Wei S., Xiang H., 2008a, China, [79]	pre- and post and follow-up (12 months) test of 30 MCP questions on knowledge (objective)& 8 questions on attitude and behaviroal intentions (subjective); during training semistructured interveiws on training method	76/78 (97) Knowledge: mean score 19.79 +/− 2.41 Self-assessed skills: Assessment: 2.77 (0.81); Policy development: 2.11 (0.69); Communication: 2.68 (0.78); Cultural competency: 2.55 (0.96); Community dimensions of practice: 2.82 (0.73); Basic PH sciences: 2.68 (0.72); Financial planning & management:2.32 (0.89); Leadership & system thinking: 2.86 (0.99).	76/78 (97) Knowledge: mean score 24.49 +/− 0.86 (*p* < 0.001 with pre-test) Self-assessed skills: Assessment: 3.69 (0.61); Policy development: 3.95 (0.51); Communication: 3.95 (0.51); Cultural competency: 3.95 (0.69); Community dimensions of practice: 3.84 (0.59); Basic PH sciences: 4.11 (0.45); Financial planning & management: 3.47 (0.82); Leadership & system thinking: 3.89 (0.55); All p < 0.05 compared with pre-test. Satisfaction: 96% thought the methods good/excellent; 100% thought the training innovative;	Knowledge: mean score 24.24 +/− 1.58 (p < 0.001 with pre-test) Self-assessed skills: Assessment: 4.35 (0.72) p < 0.05 compared with post-test; Policy development: 2.94 (0.55) p < 0.05 compared with post-test; Communication: 3.82 (0.61); Cultural competency: 3.56 (0.49); Community dimensions of practice: 3.99 (0.51); Basic PH sciences: 3.74 (0.69); Financial planning & management: 2.66 (0.74) p < 0.05 compared with post-test; Leadership & system thinking: 3.82 (0.62). All p < 0.001 compared with pre-test
	Wang C., Xiang H., 2010, China, [80]	pre- and post and follow-up (12 months) test of 30 MCP questions on knowledge (objective)& 8 questions on attitude and behaviroal intentions (subjective); during training semistructured interveiws on training method	RR 226/237(95) Knowledge: mean score 18.50 +/− 3.23 Self-assessed skills: Assessment: 2.54 (0.76); Policy development: 2.33 (1.06); Communication: 3.16 (0.84); Cultural competency: 2.26 (0.76); Community dimensions of practice: 2.69 (0.81); Basic PH sciences: 3.12 (0.93); Financial planning & management: 2.07 (1.03); Leadership & system thinking: 2.71 (0.99).	RR 226/237(95) Knowledge: mean score 22.78 +/− 1.14 (p < 0.001 with pre-test) Self-assessed skills: Assessment: 3.91 (0.65) p < 0.05; Policy development: 3.48 (0.70) p < 0.05; Communication: 4.13 (0.65) p < 0.05; Cultural competency: 3.44 (0.61) p < 0.05; Community dimensions of practice: 3.87 (0.73) p < 0.05; Basic PH sciences: 4.69 (0.49) p < 0.05; Financial planning & management: 3.26 (0.74) p < 0.05; Leadership & system thinking: 3.05 (0.69). NS Satisfaction: 92% thought training methods excellent, 96% satisfied with trainers’ performance, 89% training approach scientific and feasible, 99% very satisfied with venue, training, logistics.	RR unknown Knowledge: mean score 22.69 +/− 2.49 (p < 0.001 with pre-test) Self-assessed skills: Assessment: 4.46 (0.73) p < 0.05 with pre- and post-test; Policy development: 2.82 (0.82) NS; Communication: 4.27 (0.61) p < 0.05 with pre-test; Cultural competency: 3.21 (0.79) p < 0.05 with pre-test; Community dimensions of practice: 3.79 (0.65) NS; Basic PH sciences: 4.35 (0.54) p < 0.05 with pre-test; Financial planning & management: 2.79 (0.92) NS; Leadership & system thinking: 2.84 (0.77) NS.
	Yamada S., 2007, Hawaii/USA, [81]	Attendance logs; Post-test: course evaluation questionnaires, interviews with participants	-	RR 85/unknown Satisfaction/methodology: 83/85 thought the case appropriate to own setting; 84/85 liked group work; 80/86 thought mixed groups to be helpful for learning; 84/85 thought the case overall worthwhile as a continuing education activity. Suggestions for better case: longer time to analyze the case and prepare for the discussion on the learning issues; provide pictures. Suggestions for the facilitator: more in-depth knowledge, all members included in discussion	-
	Yellowlees P., 2008, USA, [82]	Post-test: survey with 4 questions and 5-point-Likert-scale answer options + open ended questions	-	RR 13/25(52) Satisfaction/methodology: 3.95 practicality of the program 4.5: time well spent 4.3: met the objectives.	-

*ARS* audience response system, *CBS* cross-border setting, *CPE* Continuing professional education (course satisfaction), *EPC* Education program and change (feasibility), *ETE* education, training or exercise, *NIY* New ideas and you (receptivity), *MPC* multiple choice, *NS* statistically insignificant, *PPE* personal protective equipment, *RR* response rate, *SARS* Severe Acute Respiratory Syndrom, *SSO* social system and the organization (climate), *TOT* training-of-trainers, *TTX* tabletop exercise, # = number of; − = no information available

The results were presented in line with the theoretical framework. First, factors of context, input, and process were presented; after, the outcomes per level are described, if possible, referring to the context, input, and process characteristics. In this way, both an overview is generated of characteristics of ETE in infectious disease control, their accuracy in reporting, and possible links between the context, input, and process of ETEs and the outcome.

## Results

### Literature search

In Total, 2201 unique studies were identified. After applying the in- and exclusion criteria for titles and abstracts, 186 full-texts were screened, leading to 51 inclusions. Citation screening led to the inclusion of another 11 studies. Figure 2 shows the flowchart of the search and selection process. The quality assessment resulted in seven studies with a good score for training (score ≥ 9), and 23 with a good score for the evaluation (score ≥ 9). Ten studies had a good quality score after combining the scores (score ≥ 17). All scores can be found in Additional file 4.

### Context

Five studies covered ETE in a cross-border setting, either a border region (*n* = 2), a point of entry (n = 2), or a multi-country setting aimed at international cooperation (*n* = 1). All other ETEs were in a non-cross border setting.

#### Target group

The target group of the ETE varied among studies, but was often improperly described in the studies. Examples are ‘public health leaders’, and ‘all staff of regional health departments’. Other studies specified a wide variety of professionals with different tasks in emergency preparedness or mixed public health professionals with emergency responders, university staff, and civilians. Participants’ motivations to participate are hardly derivable.

#### Recruitment & Autonomy

The majority of studies left any recruitment technique or clarified participants’ motivation unnoticed. Three studies reported mandatory participation, six studies highlighted the free choice of people participating, and two reported on freely available online courses. In Hoeppner et al., participants had to apply for participation, thereby suggesting motivation [49]. Fowkes et al. 2010 formulated their highly motivated participants as a limitation in the interpretation of their identified effectiveness of the ETE [45].

#### Training needs

In total, eleven studies performed a training needs assessment among the target population before designing the ETE. Also, training needs were obtained via literature studies, the ETE designers’ experienced-based vision [23], or by inquiry of disaster plans and local emergency management policies [55, 69]. Several studies specifically aimed to identify gaps and needs through the exercise [56, 68].

### Input

#### Training topic

The studies discussed a wide variety of ETE topics. Twenty-three studies focused on preparedness and seventeen on response. The main topics were bioterrorism (*n* = 8), a pandemic (n = 8), or a specific disease outbreak (*n* = 9), of which five focused on influenza (*n* = 5). Odd ones out were among others training on risk communication [41], leadership [64], and one health [33]. Five studies, all TOTs, incorporated didactics as a training topic.

#### Trainers

A minority of studies indicated to have competent, experienced trainers or facilitators (*n* = 18). A majority of studies described the trainers without showing their experience or competence, by generally describing them as “instructor” or “university staff”, or left trainers completely unreported (*n* = 30).

#### Development & quality of the material

The development of learning material was discussed in all but seventeen studies. Most theories were derived from constructivist learning principles, such as the Adult Learning Theory [37, 60], or problem-based learning [81]. Other used theories included the Dreyfus model [59], theory from Benner [49, 59], continuing education [28, 59], and blended learning [36]. ETEs were also based on existing competencies [37, 44, 50, 71], previously existing materials, and developers’ experience from previously performed training or exercises. The developers of the material were mostly public health professionals (*n* = 12), followed by people from universities or public health schools (*n* = 10). The help of higher departments, such as from ministry level, the national center for disease control, or the WHO, were named several times [32, 63, 74]. In two studies, graphical designers were involved in the development of realistic images or virtual environments [76, 82].

### Process

#### Classical designs

Eight studies described educational programs as part of university programs or courses, of which Yamada et al. describe an interdisciplinary and problem-based methodology during education [81], and Orfaly & Biddinger et al. and Rega et al. integrated table-top exercise in university courses [61, 67]. In the other six studies, methods were weakly described, merely referring to university programs or courses.

Nineteen studies evaluated a training of which several combined their training session with an exercise [25, 65] or real-life project [64]. Two studies left their training methodology unspecified [35, 42]. Of the other studies, all except one supported interactivity among learners or between learners and trainers by referring to interactive lectures or discussion. Detailed descriptions of training designs lacked and were restricted to summarizing words such as “using participatory methods” [31] or “an online lecture” [36, 63]. Studies delivering any detail on methodology refer to the adult learning principles, active learning, interactivity, multi-disciplinarity, or participatory methods, and explicitly away from passive methods.

Exercises were described in 24 studies, of which sixteen were table-top exercises and six simulation exercises specifically. The most common elements of table-top exercises in these studies were a lecture beforehand; a presentation of the scenario; an initial individual response; a pre-arranged and guided discussion in small, multi-disciplinary groups of local partners. Subsequently, a presentation in a larger group and a debriefing followed. Often, more than one scenario was included in the exercise. Most considerable differences between studies are the detail level of described methodology, and whether individuals, small- or large groups have to respond. Again we see more detailed study descriptions for studies that refer to the adult learning principles.

#### Innovative design - wide reach

Seven studies had a TOT design, of which three integrated the second wave of training. This second wave was delivered by the TOT participants [33, 54, 74], whereupon participants could immediately apply what was learned. All TOTs contained mixed methods. Often passive methods, such as lectures or presentations, were combined with active methodologies, such as guided discussions, clinical training, or active presenting. For two TOTs, the used ETE methodologies were largely unknown.

Seven studies studied ETE with online or new methodologies such as a virtual reality training [76], audience response system [77], the use of the intranet for training [52], e-modules [28, 31, 50], and combinations of e-learning and on-site learning [36]. Online ETEs had natural opportunities to spread the learning moments over a longer period. Also, participants were able to follow the ETE at their own pace. Some simulation exercises also used online methodologies in the form of blog websites where participants had to respond from their office to signals [21, 22, 52].

#### Innovative design - enhanced realism

Elements that were described to enhance the feeling of reality were among others the use of real work locations such as at an airport [56]; a computer simulation model generating feedback depending on participants’ decisions in a simulation exercise [26, 27]; interaction with scenario cards guiding each exercise to different possible outcomes [70]; initial ambiguity in an exercise case and drop-out of participants during the exercise [71]; moulaged or simulating patients [29]; and external consultations of experts during the exercise [75]. Rega & Fink 2013 report on a semester-long simulation exercise to keep up a realistic time frame [67].

#### Duration, interval & goals

General duration of ETEs varied between 30-min training and years-long curricula. TOTs mostly lasted several days to weeks. Educational courses lasted between 14 h and two years, training between 14 h and one year. Fifteen studies did not elaborate on the duration of the ETE. The interval and time between intervals are hardly described. The goals of ETE were addressed in most studies (*n* = 47), although often stated on the organizational level or implicitly integrated into the text instead of presenting trainable and measurable competencies. An overview of the outcomes on context, input and process are shown in Table 1.

### Evaluation & Outcome

#### System-performance

System-performance was evaluated by four studies that used participants’ evaluations of organizational achievements after the ETE [32, 38], or external evaluations [45, 48]. None of the studies assessed the system effects of ETEs in a cross-border setting. Becker et al. 2012 evaluated a postgraduate education curriculum after two years in a developing setting [32]. This curriculum impressively increased the local public health system. The three other studies (*n* = 682; 1496; unknown) evaluated several table-top- and simulation exercises. These exercises seem effective on the system level regarding improving a prepared workforce by emergency planning [45], relationships among colleagues [48], and communication systems [48]. Potter et al. 2005 did not aim to evaluate system-performance but had a coincidental finding on this level: right after the training period, a real infectious disease outbreak occurred. According to the involved professionals, the response was well managed because the members of the response team had become acquainted with each other during the training [64].

#### Behavior

Nine studies, including two TOTs [60, 62], evaluated the outcomes on a behavioral level. Evaluation of behavior was primarily timed directly after the ETE, while six studies performed an additional follow-up test. Behavioral change was mainly self-assessed by participants, leading to subjective measurements. In one study, local supervisors were appointed to assess trainees’ behavioral change [36]; another used a report on ministry level next to participants’ self-assessments [40]. No control-groups were used.

The educational curricula seem to change behavior such as initiating the updating of plans, expanding professional networks, and improving collaboration (*n* > 244). Table-tops lead, according to ministries’ reports, to increased development of further exercises and a more regular assessment of public health preparedness (*n* = unknown). Online modules had a low response rate (< 18%), but changed behavioral intentions among responding participants (*n* > 55) [28, 63]. According to local supervisors (*n* = 511), the combination of online learning and on-site training led to improved work performance. One study reported on behavioral change after table-tops in a multi-country setting but did not mention any result in interaction between countries [40]. According to Orfaly et al. 2005 and Otto et al., TOTs seem moderately effective, since 20 and 44%, respectively, conducted exercises after six months (*n* = 118; *n* = 168) [60, 62].

#### Learning – knowledge

Thirty-three studies used knowledge to evaluate the effect of an ETE, including four TOTs, and four ETEs in a cross-border setting. The majority of knowledge was evaluated in pre- and post- knowledge tests (*n* = 20) compared to self-assessments of knowledge using Likert scales. Compared to studies using knowledge tests, those using self-assessments reported more detail on how knowledge had improved. Knowledge particularly improved on organizational and functional content, such as understanding response protocols or describing functional roles or the chain of command within an organization. This is understandable since self-assessments can explicitly ask what they aim for, while knowledge tests can only provide a test score. No control-groups were used, one study compared two groups that were exposed to two different methodologies [76].

Knowledge shows a clear increase directly after ETEs. The five studies that used knowledge tests, performed follow-up tests and reported the results show a scientificly significant improved knowledge level directly after the ETE and up to 12 months after [42, 76, 78–80]. Response rates were unknown, and the duration of these ETE programs varied between fourteen hours and four weeks. Umble et al. showed equal increase in knowledge between classical education and a broadcast [76]. Regarding ETEs in a cross-border setting, all using mixed methods and clearly stated their goals, knowledge increase was shown after table-tops and training. However, these studies used self-assessments or unknown scoring methodologies.

#### Learning – skills

Twenty-one studies evaluated an ETE on skills, including three TOTs but none in a cross-border setting. Practiced skills vary from a majority of organizational, communicational, team, and leadership skills, to a minority of more medical skills such as surveillance or the use of personal protective equipment. Except for one study using skill demonstrations [74], most studies performed self-assessment of improvements comparing pre- and post-tests. Seven studies also performed a follow-up test.

According to participants’ self-assessments, all ETEs were effective skill-builders. A statistically significant increase in skills is shown for training, while this outcome remains insignificant for most tabletop- and simulation exercises. Follow-up evaluations indicated even a further increase in skills in the period after the ETE, although these results are self-assessed and mainly statistically insignificant. Two TOTs showed a significant increase in planning, implementation, and evaluation after a table-top exercise [33, 54]; follow-up results were unavailable here.

#### Learning – attitude

Fifteen studies reported on a change in attitude, including one for a TOT [43], and one for several table-tops in a cross-border setting [40]. The evaluated attitudes comprised the awareness of and motivation to develop future preparedness plans and programs, or an increase in confidence. We saw mainly training and exercises evaluating attitude. Attitude was assessed by rating statements.

We saw a sustainable change in attitude directly and 1–3 months after both online and face-to-face training. These training programs lasted between 1,5 and 14 h but had unclear methods. Table-top exercises varied in their capability to change attitude, since both significant change [72, 75] and fairly indifference [34, 71] was shown, indicating that more detailed evaluation is required. The table-tops in a cross-border setting seemed to enhance participants’ motivation to develop and exercise programs [40]. Dickmann et al. 2016 reported a relation between knowledge and attitude: participants with higher knowledge also had congruent confidence levels to respond and advocate for change [41]. Data regarding TOTs do not suffice aggregation of results.

#### Reaction

Forty-five studies assessed ETE on the reaction level, mostly by participants rating statements on satisfaction and methodology using Likert scales, directly after the ETE. The ETEs in crossborder settings show high satisfaction among participants regarding table-tops and simulation exercises. One TOT showed satisfied participants of the second wave of training. We will present the results for different designs.

Training programs scored satisfactorily directly after the training, despite the substantial differences in design: after a 30-min pandemic preparedness training [46], 98% of participants thought the program valuable, as thought 95% after several face-to-face modules on emergency preparedness [44], and 92–96% after a preparedness training of 14 days [78, 80]. Remarkably, the one study performing a follow-up test identifies the lowest satisfaction of all training programs, with a mean score of 4/5 after a 2-day Zika response training [35].

Only one study evaluated reaction after an exercise with a follow-up test [40], all others were restricted to post-tests. Table-top exercises overall scored high on satisfaction, mainly based on their potential to practice together (77% agreed [34]), to build relationships (80–90% agreed [58]); to improve emergency or contingency planning (73% agreed [34]); and to identify gaps (89% [62] and 77% [58] agreed). Biddinger et al. identified higher satisfaction among regional exercise respondents compared with single institution respondents regarding their understanding of agencies’ roles and responsibilities (*p* < 0.001), engagement in the exercise (*p* = 0.006), and satisfaction with the combination of participants (p < 0.001) [34]. The right combination of participants was in several studies scored as one of the most valuable aspects. A disadvantage of table-top exercises was the lack of identification of key gaps in individuals’ performance [40]. Further made recommendations for exercises were: to clearly formulate specific objectives; to be as realistic as possible; to ground practical response in theory; to be designed around issue-areas rather than scenarios; to have a forced, targeted and time delineated discussion and decision making; to have limited number of participants but to include all key perspectives and especially leadership perspectives; to be collaboratively designed and executed with representatives from participating agencies, external developers, and facilitators; to have networking possibilities; and to use trained evaluators.

Simulation exercises were less assessed on reaction, and outcomes show a slightly lower satisfaction than the table-top exercises. However, in three studies, “most participants” or over 80% of participants still agreed on their readiness being increased by simulation. The full-scale simulation at an airport stresses the need for specific goals, in this way preventing deprioritizing the public health response by trying to test everything at the same time [56]. Also, it is paramount to have clear roles and responsibilities of the various agencies involved, and to have all required capacity available [56]. One study showed a positive relationship between the duration and the contact and communication between health departments after a joint exercise [22].

Ten studies reported reaction directly after innovative methodologies. Several studies added online blogs, pages, or systems to a simulation exercise [22], a lecture [50], or a combination of classical designs. Other studies evaluated pure technologies such as an audio-response system [77], or a virtual reality environment [82]. For innovative methods, satisfaction was generally high, although technical issues were often reported. For example, the e-modules in Baldwin et al. were launched via the intranet of a public health organization [30], thereby benefitting from high accessibility but facing extensive, unforeseen updates, a rigidness for change and delayed updated because ownership was not designated. The VR environment exercise met its objectives and was time well spent, but the participants and authors suggest further technology innovations before this method can be used at large scale [82]. An overview of all outcomes, including those not mentioned above [24, 39, 47, 51, 53, 57, 66, 73], are shown in Table 2.

## Conclusions

This study aimed to review the different ETE methodologies that are used by professionals in infectious disease management, how these methodologies are evaluated, and what their effect is. We have a particular focus on cross-border settings, such as POEs, and methodologies with a wide reach. We identified various types of ETEs – from nationwide online preparedness programs till the hands-on local trainings during an outbreak - but with generally few details on the exact methodology. Both the lack of details and the predominance of short-term and subjective evaluations impede conclusions on what methods in which settings lead to both positive and sustainable outcomes. Our results point out the need for standardized evaluations, preferably with a long-term scope, that are shared among trainers and organizers. We developed a theoretical framework that can be used to structure future evaluations. These evaluations, then, will hopefully not only inspire future developers to come up with successful ETE designs but also lead to recommendations for the best exercise-effect ratio.

Reports on system and behavioral level outcomes are scarce, leaving us with a majority of lessons learned on lower outcome levels as learning and reaction. While the convincing and sustainable increase in knowledge and skills are hopeful indications for system improvements and support the use of ETEs for learning, several intervening factors are possible. Among others, evaluation tests in itself are one of the most sustainable learning techniques [18]. The knowledge tests and demonstrations activate knowledge and skills and might be responsible for the effect. Also, control groups are missing while often immediate causes are seen for the organization of an ETE, such as a growing pandemic or a recent bioterrorism attack. These events require ETEs, but might also lead to greater attention for and learning about the subject despite any ETE. We saw a learning effect that increases during follow-up and is independent of ETE duration, which is further supporting this confounding effect.

While cross-border infectious disease control receives international attention, and in Europe alone, almost all countries have designated POE to be prepared to handle cross-border health threats, we identified only five studies describing an ETE evaluation in a cross-border setting. This is too low a number to draw general conclusions about the effectiveness of ETEs in a cross-border setting. Findings from ETEs in general infectious disease management should be used for this setting untill more specific evaluations are available. However, one crucial difference between the cross-border and non-cross-border setting is the larger and more diverse set of stakeholders that are involved in cross-border settings. Not only several countries are involved, but also information and cooperation are needed between general public health, health professionals, and specific port, airport and ground-crossing officials. While the studies in cross-border settings did not elaborate on their specific settings, many other studies in our review identified a strengthened network, better knowledge of roles & responsibilities, and enhanced relations among the most valuable aspects of training and exercises. In other disciplines, it was also discovered that sharing the same language [83], the focus on relationships, and collaborative management skills [84] are essential factors of collaborative learning. We consider these findings as prudent support for training and exercising in cross-border settings.

Because cross-border health threat prevention requires collaboration between countries and a shared minimum level of functioning, we considered TOT approaches and online methodologies for their potential to reach several locations and a large audience at once. Both methodologies have potential and some remaining challenges. TOTs seem as effective in learning as other training methods, and their participants are satisfied. Unfortunately, TOTs are only moderately effective regarding their principal goal: the organization and delivery of future ETEs. In this way, the potential exponential increase in delivered training sessions and trainees compared to single direct training remains limited. To reach their potential, the barriers that TOT participants perceive, such as a lack of confidence, time or resources for ETE delivery, or other priorities during their duties, should be taken seriously in future TOT programs. Online methodologies overcome specific barriers that were identified for TOT approaches. Both in our study as in another recent review on undergraduate medical education indicate that online learning “enhances knowledge and skills”, while evidence is lacking “that offline learning works better” [85]. However, technical issues and a lack of ownership of the online environments are remaining barriers. Also, we only had a low number of studies to evaluate. We call for more, enhanced evaluation of ETEs using innovative and online methods, which is stressed recently by other reviews and the WHO [86, 87].

This review has several strengths and limitations. First, we restricted our analysis to what was available in the peer-reviewed literature databases and did not study the body of grey literature. Although it is very probable that more evaluations are performed, orienting searches in the grey literature yielded a limited amoung of evaluations, indicating that the majority of ETEs in a crossborder setting are not made public. However, the theoretical framework developed in this study can be used on a wide variety of ETEs, including those not publicly available within public health organizations. Furthermore, this theoretical framework can be used to support the design and evaluation of ETEs, and a more complete reporting in the peer-reviewed literature.

Second, the didactic scope of our review can be seen both as a limitation and a strength. The collaborative evaluation of education, training and exercising leads to broad and generic conclusions possibly limiting conclusions on individual ETEs whose goals widely vary among each other. However, restricting our results to either education, training or exercising specifically is also problematic. Although general distincitions are possible, on an individual level these are often arbitrary. As our results show, exercises are often taken as part of training or educational programs. For example, organization-wide exercises are used for training on the system level – is an organization prepared to respond effectively -, but training is also used for the handling of individual patients. We, therefore, chose to evaluate all three ways of learning, using the same four levels of evaluation. In this way, the results in this study do not only display the effect of the ETEs, but also specify these to the evaluation level.

Thirdly, we restricted our focus on infectious disease prevention and control in a public health setting. While public health responses to chemical, radiological or nuclear threats demand another set of professionals, they share many of the aspects of contamination and could be included in future reviews. The theoretical framework as developed in this study, may be well applicable to use for evaluations in these adjacent disciplines. We consider it a strength that, to the best of our knowledge, this is the first attempt to assess ETEs in infectious disease control systematically. In addition to previous efforts [9], we studied evaluations and outcomes with greater detail and with the comprehensive framework we developed, we have contributed to the body of knowledge regarding the performance of systematic reporting and evaluation of ETEs.

Future studies should focus on the development of a standardized evaluation format integrating details of context, input, and process and suggesting planning and questionnaires for evaluations. Future training developers should first focus on the formulation of clear ETE goals, then attach the required outcome level and subsequently choose the appropriate evaluation methods. For example, if one intends to improve an airport’s capability to prevent secondary transmissions during a case of tuberculosis on a plane, then this goal is formulated on the system level. However, if the goals on the system level are not met, the formulation of goals and evaluations of outcomes on individual behavior, knowledge, skills are required to what and who should be further supported. Choosing the appropriate evaluation methods might involve requesting access to track-records, including external observators, planned skill demonstration, or validated knowledge tests. Lastly, we highly recommend sharing evaluations and lessons learned of ETEs on a broad scale to directly support co-organizers and provide policymakers with the chance to deploy costs, time, and capacity towards the optimal effect. By standardizing the evaluation of ETEs, comparisons with methods in general adult education would become possible and provide an even broader base for recommendations on effect. We call for international efforts to facilitate this sharing of evaluations and experience, for example, through maintaining a sustainable electronic training platform where standard information can be registered, and exchanged about the set-up, implementation and evaluation of ETEs. A standard set of scenarios in cross-border setting or training materials could further encourage this development. Previous time-restricted projects have shown their potential [88], but a sustainable option has been missing.

We conclude that although extensive training and education programs exist in infectious disease control, recent literature can only partly and prudently prove their added value, especially in cross-border settings. We see promising results for online methodologies reporting similar results as offline training, although relationship building and networking are among the aspects most valued by participants of face-to-face training. Above all, future developers of ETEs should not forget the long-term perspective of their efforts; sharing the evaluations benefits a crowd of colleague organizers from detailed and thorough reporting and evaluation. This paper, therefore, presents a call for publishing ETE evaluations in order to facilitate overall system learning and preparations of a workforce that can cope with the perpetual challenges of global infectious disease control.

## Supplementary information

**Additional file 1.** Theoretical background. Theoretical background of the data analysis of the review; extended version as compared to the section presented in the article .

**Additional file 2.** Search syntax. The search syntax used for this literature review.

**Additional file 3.** Quality Assessment form. The form composed for quality assessment of the studies; the first part assessing the quality of the education, training or exercise method and the second part that of the performed scientific study.

**Additional file 4.** Results Quality Assessment. The results of the quality assessment are shown here.

## Data Availability

The datasets used and/or analyzed during the current study are available from the corresponding author on reasonable request.

## Abbreviations

ETE: Education, training, or exercise
TOT: Training-of-trainers
POE: Point of entry: a port, airport or ground-crossing
