# Supplementary material for: Educating, training, and exercising for infectious disease control with emphasis on cross-border settings: an integrative review
Source: Global Health. 2020 Sep 3;16:78. doi: 10.1186/s12992-020-00604-0 (PMC7468091; doi:10.1186/s12992-020-00604-0)
Supplement: Supplementary file 1 — Additional file 1. Theoretical background. Theoretical background of the data analysis of the review; extended version as compared to the section presented in the article . [file 12992_2020_604_MOESM1_ESM.pdf]

Additional file 1 – Theoretical Backround

To study the effectiveness of training activities, we used the the seminal model of Kirkpatrick [1], which contains four levels with gradual importance and complexity of evaluation. On the individual level, it covers trainees' satisfaction, competence, and behavioral change, and at system level we face performance of the organization or even larger system [1, 2]. Kirkpatrick suggests in his study in 1996 that all four levels should be assessed for a proper ETE evaluation. This might not always be possible, due to time, financial or capacity problems [1]. Especially the results which will turn out after a certain period, behavior change, system change, are hard to measure. Lower levels of effectiveness might be indicators of these higher levels, although it remains unclear to what extent.

Other evaluation models added several aspects to the Kirkpatrick model that influence effectiveness and that need to be assessed to conclude what exactly was effective. The CIRO model, for example, describes the influence of the context where the learning takes place, the IPO model explains the input of facilitators'experience, prior education, the quality of the material, and the design and instructional strategies [2-4]. In this review, variables of context, input, process and outcome will be distracted to link these characteristics with effect.

Aside from how effectiveness of training activities can be measured, we also incorporated the perspective of the individual participant and how they learn best. A significant amount of theories on the psychology of learning, behavioral change and motivation exist. In professional education, the Adult Learning Theory is currently most prominent. This theory is based on the constructivist view that knowledge is produced in the mind, linked to prior knowledge, and tested in interaction with fellows. To link new information during education or trainings to prior knowledge and experience, and to facilitate social interaction among trainees is paramount [5, 6]. Many aspects of this Adult Learning Theory are in line with the motivational Theory of Self-Determination [5], according to which adults are intrinsically motivated if they have a choice to learn (autonomy), they consider themselves able to learn (mastery or competence), and they know why they would learn (purpose or relatedness) [7].

If all conditions of the Adult Learning Theory and Self-Determination Theory are met, one can still question how sustainable this learning is. Measuring increased knowledge levels right after a lecture will be of no surprise. However, what the effect of this lecture is after several months is less clear. To get insight into this question, Dunlosky et al. performed a literature review on learning techniques for individuals and the sustainability of their effect specified for different target groups [8]. Most sustainable techniques for professionals in infectious disease control, people with high prior knowledge on a subject, that require problem-solving and comprehension in their job seem 'practice testing' and 'distributed practice'. Practice testing implies performing a test of the learned material after some time and distributed practice implies that learning time is distributed in small blocks over a longer period instead of one longer learning opportunity. This might have implications for different used ETE methodologies. Practice testing can be identified during case-studies or exams, and distributed practice refers to the sequal of performed ETEs.

References:

1. Kirkpatrick D. Great ideas revisited: Revisiting Kirkpatrick's Four-level model. Training & Development 1996: Volume unknown; 54-7.

2. Reio TG, Rocco TS, Smith DH, Chang E. A Critique of Kirkpatrick's Evaluation Model. New Horizons in Adult Education & Human Resourse Development 2017: 29(2); 35-53.
3. Bushnell DS. Input, Process, Output: a Model for Evaluating Training. Training & Development Journal, March 1990: 41-3.
4. Barden V. Book review: Peter Warr, Michael Bird & Neil Rackham: Evaluation of Manamgement Training, 1970, Londen, Gower Press, 112pp, £2.50.
5. Taylor DCM, Hamdy H. Adult learning theories: implications for learning and teaching in medical education: AMEE Guide No. 83. Medical Teacher 2013: 35(11); 1561-72.
6. Discroll MP. Psychology of learning for instruction. Harlow: Pearson Education Limited; 2014.
7. Ryan RM, Deci EL. Self-determination theory and the faciliation of intrinsic motivation, social development, and well-being. American Psychologist 2000;55(1):68-78.
8. Dunlosky J, Rawson KA, Marsh EJ, Nathan MJ, Willingham DT. Improving students' learning with effective learning techniques: promising directions from cognitive and educational psychology. Psycyological science in the public interest 2013: 14(1);4-58.
