# Supplementary material for: Educating, training, and exercising for infectious disease control with emphasis on cross-border settings: an integrative review
Source: Global Health. 2020 Sep 3;16:78. doi: 10.1186/s12992-020-00604-0 (PMC7468091; doi:10.1186/s12992-020-00604-0)
Supplement: Supplementary file 2 — Additional file 2. Search syntax. The search syntax used for this literature review. [file 12992_2020_604_MOESM2_ESM.pdf]

Additional File 2 – Search Syntaxes.

Search strategy per database:

File	Number of studies
Medline 20180924	541
Embase 20180927	943
Cinahl 20180928	221
Psycinfo 20180928	189
Eric 20180928	132
WOS 20180928	705

Medline

Database: MEDLINE <1946 to Present, updated daily>
Search Strategy:
----
1 "Planning and preparing for public health threats at airports".fc_titl. (1)
2 "A simulator-based nuclear reactor emergency response training exercise".fc_titl. (1)
3 "4.Effectiveness of educational outreach in infectious diseases management: a cluster randomized trial in Uganda".fc_titl. (0)
4 "Effectiveness of educational outreach in infectious diseases management".fc_titl. and "2016".fc_pubyr. (1)
5 "Training of public health personnel in handling CBRN emergencies".fc_titl. and "2014".fc_pubyr. (1)
6 "Immersive simulation education".fc_titl. and "2014".fc_pubyr. (1)
7 "7.Improving emergency preparedness system readiness through simulation and interprofessional education".fc_titl. and "2014".fc_pubyr. (0)
8 "miller$".fc_auts. and "emergency preparedness system readiness through simulation ".fc_titl. (1)
9 1 or 2 or 4 or 5 or 6 or 8 (6)
10 "prevention and control training and capacity building during the Ebola epidemic in Guinea".fc_titl. (1)
11 9 or 10 (7)
12 from 11 keep 1-7 (7)
13 "setting infectieziekten of hazards".ti. (0)
14 exp Public Health/ (7094060)
15 ((public adj3 health) or (communit* adj3 health)).tw. (243016)
16 ((public adj3 health) or (communit* adj3 health)).kf. (42052)
17 (preventive adj3 medicine).tw. (6020)
18 ((infecti* adj3 prevent* adj3 contr*) or ipc).tw. (5757)
19 ((infecti* adj3 prevent* adj3 contr*) or ipc).kf. (2543)
20 (surveill* adj3 transm* adj5 disease*).tw. (106)
21 (surveill* adj3 transm* adj5 disease*).kf. (1)
22 Pandemics/ (4452)
23 Disease Outbreaks/ (74196)
24 (pandemic? or (disease adj3 outbreak?)).tw. (30230)
25 (pandemic? or (disease adj3 outbreak?)).kf. (1351)
26 (infect* adj3 disease* adj8 (transm* or manag*)).tw. (5126)
27 (infect* adj3 disease* adj8 (transm* or manag*)).kf. (180)
28 or/14-27 (7224997)
29 "Hazardous Substances"/ (8209)

30 "Occupational Exposure"/ (50645)
31 exp Radiation Exposure/ (67320)
32 disaster planning/ or mass casualty incidents/ (14155)
33 Civil Defense/ (2547)
34 ((radioactive or chemical) adj3 hazard?).tw. (1165)
35 biohazard*.tw. (687)
36 exp biohazard release/ or exp chemical hazard release/ or exp radioactive hazard release/ (7039)
37 or/29-36 (143717)
38 28 or 37 (7244536)
39 from 38 keep 1 (1)
40 "onderdeel cross border".ti. (0)
41 automobile driving/ or exp travel/ (41293)
42 "Emigration and Immigration"/ (24451)
43 exp transportation/ or exp aviation/ or exp motor vehicles/ or exp railroads/ or exp ships/ (64537)
44 (crossborder or "cross-border" or bordercross* or "border-cross" or "border crossing" or "border crossings" or "ground-cross" or "ground crossing" or "ground crossings" or groundcross* or "point of entry" or "points of entry" or transport or transfer or airport* or aeroport* or plane or port or ports or harbors or harbours or ship* or boat or boats or vessel * or craft* or vehicle* or car or cars or bus or coach or conveyanc*).tw. (994719)
45 (crossborder or "cross-border" or bordercross* or "border-cross" or "border crossing" or "border crossings" or "ground-cross" or "ground crossing" or "ground crossings" or groundcross* or "point of entry" or "points of entry" or transport or transfer or airport* or aeroport* or plane or port or ports or harbors or harbours or ship* or boat or boats or vessel * or craft* or vehicle* or car or cars or bus or coach or conveyanc*).kf. (36553)
46 airport?.tw. (2306)
47 airport?.kf. (75)
48 (port adj5 health).tw. (148)
49 (port adj5 health).kf. (3)
50 travel*.tw. (54792)
51 travel*.kf. (2114)
52 (border* or cross?border*).tw. (105499)
53 (border* or cross?border*).kf. (2162)
54 or/41-53 (1234105)
55 11 and 54 (2)
56 "onderdeel educatie".ti. (0)
57 ed.fs. (258610)
58 exp Health Personnel/ed (55295)
59 inservice training/ or staff development/ (27567)
60 education/ or education, professional/ or exp education, continuing/ or education, public health professional/ or mentoring/ or preceptorship/ or teacher training/ (86906)
61 exp Public Health/ed [Education] (7883)
62 (train* or supervis* or course* or hands?on or didacti* or exercis* or educat* or program* or skill* or curricul*).tw. (2270963)
63 (train* or supervis* or course* or hands?on or didacti* or exercis* or educat* or program* or skill* or curricul*).kf. (129835)
64 Capacity Building/ (1900)
65 (capacit* adj3 build*).tw. (6060)
66 (capacit* adj3 build*).kf. (539)
67 or/57-66 (2435798)
68 38 and 67 (1013746)

69 (international adj5 (travel* or transport*)).tw. (2379)
70 (international adj5 (travel* or transport*)).kf. (50)
71 54 or 69 or 70 (1234160)
72 11 and 71 (2)
73 68 and 71 (48496)
74 tm.fs. (137056)
75 transm*.tw. (563494)
76 transm*.kf. (19113)
77 74 or 75 or 76 (642987)
78 73 and 77 (2590)
79 exp Evidence-Based Practice/ (81564)
80 (train* adj8 evaluat*).tw. (21010)
81 (pre?test* or post?test*).tw. (18126)
82 (educat* adj5 (result* or outreach or improv*)).tw. (32463)
83 ((transfer* adj3 skill*) or (improve adj3 skill*)).tw. (4966)
84 Program Evaluation/ (57444)
85 (program adj3 (evaluat* or effect* or appropiat*)).tw. (20514)
86 Program Evaluation/ (57444)
87 ((transfer* adj3 skill*) or (improve adj3 skill*)).tw. (4966)
88 (educat* adj5 (result* or outreach or improv*)).tw. (32463)
89 Quality Improvement/ (17897)
90 ((quality adj5 improv*) or (best adj3 practic*)).tw. (171117)
91 ((quality adj5 improv*) or (best adj3 practic*)).kf. (5028)
92 or/79-91 (388202)
93 73 and 92 (4974)
94 77 and 93 (191)
95 international cooperation/ or medical missions, official/ (44947)
96 international.ti. (66867)
97 international.kf. (11880)
98 Population Health/ (254)
99 (population adj3 health).ti. (3969)
100 (population adj3 health).kf. (785)
101 64 or 65 or 66 or 69 or 70 or 77 or 95 or 96 or 97 or 98 or 99 (762533)
102 or/57-63 (2433345)
103 38 and 54 and 92 and 102 and 101 (432)
104 11 and 38 (7)
105 11 and 54 (2)
106 11 and 67 (7)
107 11 and 92 (3)
108 11 and 102 (7)
109 101 and 11 (3)
110 11 and 38 and 67 and 102 (7)
111 11 and 38 and 67 and 102 and (54 or 92 or 101) (5)
112 11 not 111 (2)
113 38 and 67 and 102 and 92 (105704)
114 113 and (54 or 101) (9956)
115 exp *Public Health/ (1648533)
116 ((public adj3 health) or (communit* adj3 health)).ti. (67350)
117 (preventive adj3 medicine).ti. (2961)
118 ((infecti* adj3 prevent* adj3 contr*) or ipc).ti. (600)
119 (surveill* adj3 transm* adj5 disease*).ti. (43)
120 *Pandemics/ (2818)

121 *Disease Outbreaks/ (48368)
122 (pandemic? or (disease adj3 outbreak?)).ti. (10530)
123 (infect* adj3 disease* adj8 (transm* or manag*)).ti. (886)
124 16 or 19 or 21 or 25 or 27 or 115 or 116 or 117 or 118 or 119 or 120 or 121 or 122 or 123 (1718599)
125 *"Hazardous Substances"/ (5635)
126 *"Occupational Exposure"/ (36591)
127 exp *Radiation Exposure/ (50583)
128 *disaster planning/ or *mass casualty incidents/ (10933)
129 *Civil Defense/ (2024)
130 ((radioactive or chemical) adj3 hazard?).ti. (276)
131 biohazard*.ti. (164)
132 exp *biohazard release/ or exp *chemical hazard release/ or exp *radioactive hazard release/ (5274)
133 124 or 125 or 126 or 127 or 128 or 129 or 130 or 131 or 132 (1743339)
134 11 and 133 (7)
135 114 and 133 (3994)
136 54 or 32 or 33 or 34 or 35 or 36 (1256305)
137 "extra voor outcome".ti. (0)
138 ((positive adj3 result*) or (deal* adj3 prepar*) or (cost-effic* adj3 train*)).tw. (79311)
139 (emergenc* adj3 respon*).tw. (5624)
140 92 or 138 or 139 (470133)
141 38 and 133 and 136 and 102 and 140 (2913)
142 141 and 101 (346)
143 141 and 11 (4)
144 11 not 143 (3)
145 11 and 102 (7)
146 11 and 140 (6)
147 11 not 146 (1)
148 (train* adj3 result*).tw. (12439)
149 140 or 148 (480041)
150 11 and (140 or 148) (7)
151 133 and 102 and 149 and 101 (2747)
152 151 and 11 (3)
153 133 and 102 and 149 (33041)
154 153 and 136 (2991)
155 154 and 11 (4)
156 11 not 101 (4)
157 (emergenc* or (pandemic adj3 manag*)).ti,kf. (106288)
158 101 or 157 (865308)
159 133 and 102 and 149 and 158 (3579)
160 159 and 11 (6)
161 157 or 64 or 65 or 66 or 69 or 70 (115661)
162 153 and 161 (1287)
163 162 and 11 (6)
164 11 not 163 (1)
165 (emergenc* or (pandemic adj3 (prepar* or manag*))).ti,kf. (106626)
166 101 or 165 (865582)
167 102 and 133 and 149 and (161 or 165) (1296)
168 167 and 11 (7)
169 167 (1296)
170 limit 169 to yr="2010 -Current" (853)

171 170 and 124 (736)
172 169 and 124 (1102)
173 (54 or 157 or 67) and 153 (33041)
174 173 and 124 (32468)
175 from 172 keep 1000-1069 (70)
176 133 and 136 and 102 and 140 (2913)
177 133 and 54 and 102 and 140 (2015)
178 177 and 124 (1961)
179 41 or 43 or 44 or 45 or 52 or 53 (1176608)
180 11 and 179 (2)
181 133 and 179 and 102 and 140 (1676)
182 133 and 102 and 149 and (161 or 165 or 179) (2909)
183 59 or 61 or 62 or 63 (2317901)
184 182 and 183 (2867)
185 *Capacity Building/ (988)
186 (capacit* adj3 build*).ti. (1373)
187 (international adj5 (travel* or transport*)).ti. (630)
188 66 or 70 or 185 or 186 or 187 (2919)
189 133 and 183 and 149 and (188 or 165 or 179) (2647)
190 189 and 124 (2435)
191 exp *Evidence-Based Practice/ (30848)
192 191 or 80 or 81 or 82 or 83 or 87 or 88 (104799)
193 192 or 138 or 139 or 191 (188591)
194 11 and 193 (5)
195 11 and (193 or 79) (5)
196 11 and 149 (7)
197 196 not 195 (2)
198 "*Preparedness".kw. (306)
199 epidemics/pc (868)
200 pandemics/pc (1038)
201 Public Health/ (73926)
202 16 or 19 or 21 or 25 or 27 or 116 or 117 or 118 or 119 or 120 or 121 or 122 or 123 (164096)
203 *Public Health/ (47162)
204 202 or 203 (182848)
205 11 and 204 (6)
206 11 not 205 (1)
207 190 and 204 (502)
208 207 and 11 (6)
209 204 and 102 and 140 and (54 or 188 or 165 or 179) (539)
210 209 and 11 (5)
211 11 not 210 (2)
212 11 and (54 or 188 or 165 or 179) (7)
213 212 and 140 (6)
214 11 not 213 (1)
215 (test adj3 scor*).tw. (18467)
216 204 and 102 and (140 or 215) and (54 or 188 or 165 or 179) (541)
217 11 and 216 (6)
218 11 not 217 (1)
219 92 or 138 or 139 or 215 (486738)
220 204 and 102 and 219 and (54 or 188 or 165 or 179) (541)= **)= setting focus + educatie + outcome + (cross border or preparedness or capacity building of international)**

Embase

Database: Embase <1974 to 2018 September 26>
Search Strategy:
----
1 exp public health/ (151371)
2 ((public adj3 health) or (communit* adj3 health)).tw. (277340)
3 ((public adj3 health) or (communit* adj3 health)).kw. (21466)
4 exp preventive medicine/ (23599)
5 (preventive adj3 medicine).tw. (8702)
6 ((infecti* adj3 prevent* adj3 contr*) or ipc).tw. (8099)
7 ((infecti* adj3 prevent* adj3 contr*) or ipc).kw. (514)
8 (surveill* adj3 transm* adj5 disease*).tw. (118)
9 (surveill* adj3 transm* adj5 disease*).kw. (2)
10 exp disease surveillance/ (23321)
11 exp communicable disease/ (20094)
12 pandemic/ (9333)
13 epidemic/ (92098)
14 (pandemic? or (disease adj3 outbreak?)).tw. (34532)
15 (pandemic? or (disease adj3 outbreak?)).kw. (3838)
16 (infect* adj3 disease* adj8 (transm* or manag*)).tw. (6356)
17 (infect* adj3 disease* adj8 (transm* or manag*)).kw. (312)
18 or/1-17 (540257)
19 hazard/ or dangerous goods/ or dangerous goods transport/ or electric hazard/ or hazard assessment/ or exp hazardous waste/ or exp health hazard/ or occupational exposure/ or occupational hazard/ or radiation hazard/ (610973)
20 exp radiation exposure/ (175491)
21 disaster planning/ (11644)
22 mass disaster/ (2153)
23 civil defense/ (1384)
24 ((radioactive or chemical) adj3 hazard?).tw. (1449)
25 biohazard*.tw. (852)
26 biosafety/ (5041)
27 or/19-26 (793339)
28 18 or 27 (1303817)
29 "onderdeel setting".ti. (0)
30 management/ (48045)
31 exp capacity building/ (3651)
32 "Planning and preparing for public health threats at airports".fc_titl. (1)
33 "A simulator-based nuclear reactor emergency response training exercise".fc_titl. (1)
34 "Effectiveness of educational outreach in infectious diseases management".fc_titl. and "2016".fc_pubyr. (1)
35 "Training of public health personnel in handling CBRN emergencies".fc_titl. and "2014".fc_pubyr. (1)
36 "Immersive simulation education".fc_titl. and "2014".fc_pubyr. (1)
37 "miller$".fc_auts. and "emergency preparedness system readiness through simulation ".fc_titl. (1)
38 "prevention and control training and capacity building during the Ebola epidemic in Guinea".fc_titl. (1)
39 or/32-38 (7)
40 28 and 39 (7)
41 "onderdeel cross border".ti. (0)

42 exp "traffic and transport"/ (192230)
43 exp migration/ (41464)
44 (crossborder or "cross-border" or bordercross* or "border-cross" or "border crossing" or "border crossings" or "ground-cross" or "ground crossing" or "ground crossings" or groundcross* or "point of entry" or "points of entry" or transport or transfer or airport* or aeroport* or plane or port or ports or harbors or harbours or ship* or boat or boats or vessel * or craft* or vehicle* or car or cars or bus or coach or conveyanc*).tw. (1120587)
45 (crossborder or "cross-border" or bordercross* or "border-cross" or "border crossing" or "border crossings" or "ground-cross" or "ground crossing" or "ground crossings" or groundcross* or "point of entry" or "points of entry" or transport or transfer or airport* or aeroport* or plane or port or ports or harbors or harbours or ship* or boat or boats or vessel * or craft* or vehicle* or car or cars or bus or coach or conveyanc*).kw. (106983)
46 airport?.tw. (3009)
47 airport?.kw. (249)
48 (port adj5 health).tw. (178)
49 (port adj5 health).kw. (9)
50 travel*.tw. (68100)
51 travel*.kw. (4076)
52 (border* or cross?border*).tw. (137532)
53 (border* or cross?border*).kw. (7342)
54 or/42-53 (1493970)
55 capacity building/ or management/ (51637)
56 (capacit* adj3 build*).tw. (7578)
57 (capacit* adj3 build*).kw. (829)
58 (international adj5 (travel* or transport*)).tw. (3417)
59 (international adj5 (travel* or transport*)).kw. (103)
60 population health/ (665)
61 (population adj3 health).ti. (4292)
62 (population adj3 health).kw. (1077)
63 (population adj3 health).kw. (1077)
64 international cooperation/ (49847)
65 or/55-64 (114427)
66 28 and 54 and 65 (2014)
67 exp education/ (1278947)
68 (train* or supervis* or course* or hands?on or didacti* or exercis* or educat* or program* or skill* or curricul*).tw. (2899706)
69 (train* or supervis* or course* or hands?on or didacti* or exercis* or educat* or program* or skill* or curricul*).kw. (207308)
70 exp evidence based practice/ (1063826)
71 (train* adj8 evaluat*).tw. (29367)
72 (pre?test* or post?test*).tw. (22286)
73 (educat* adj5 (result* or outreach or improv*)).tw. (51737)
74 educat*.kw. (66801)
75 ((transfer* adj3 skill*) or (improve adj3 skill*)).tw. (7176)
76 program evaluation/ (11164)
77 health program/ (101953)
78 (program adj3 (evaluat* or effect* or appropiat*)).tw. (27425)
79 ((transfer* adj3 skill*) or (improve adj3 skill*)).tw. (7176)
80 (educat* adj5 (result* or outreach or improv*)).tw. (51737)
81 total quality management/ (51076)
82 ((quality adj5 improv*) or (best adj3 practic*)).tw. (248912)
83 or/67-82 (4575499)

84 66 and 83 (862)
85 or/70-82 (1543432)
86 67 or 68 or 69 (3552339)
87 66 and 85 and 86 (185)
88 from 84 keep 1-824 (824)
89 public health/ (151371)
90 from 88 keep 1-824 (824)
91 *public health/ (53501)
92 ((public adj3 health) or (communit* adj3 health)).ti. (56848)
93 ((public adj3 health) or (communit* adj3 health)).kw. (21466)
94 exp *preventive medicine/ (9751)
95 (preventive adj3 medicine).ti. (2164)
96 ((infecti* adj3 prevent* adj3 contr*) or ipc).ti. (798)
97 ((infecti* adj3 prevent* adj3 contr*) or ipc).kw. (514)
98 (surveill* adj3 transm* adj5 disease*).ti. (45)
99 (surveill* adj3 transm* adj5 disease*).kw. (2)
100 exp *disease surveillance/ (4633)
101 exp *communicable disease/ (9235)
102 *pandemic/ (2276)
103 *epidemic/ (29521)
104 (pandemic? or (disease adj3 outbreak?)).ti. (11767)
105 (pandemic? or (disease adj3 outbreak?)).kw. (3838)
106 (infect* adj3 disease* adj8 (transm* or manag*)).ti. (985)
107 (infect* adj3 disease* adj8 (transm* or manag*)).kw. (312)
108 or/91-107 (169812)
109 39 and 108 (5)
110 108 and 83 (58002)
111 (test adj3 scor*).tw. (25906)
112 ((positive adj3 result*) or (deal* adj3 prepar*) or (cost-effic* adj3 train*)).tw. (121471)
113 (emergenc* adj3 respon*).tw. (6964)
114 70 or 71 or 72 or 73 or 75 or 76 or 78 or 79 or 80 or 82 or 111 or 112 or 113 (1531402)
115 110 and 114 (12404)
116 capacity building/ (3651)
117 (capacit* adj3 build*).ti. (1611)
118 *capacity building/ (917)
119 (capacit* adj3 build*).kw. (829)
120 (international adj5 (travel* or transport*)).ti. (704)
121 (international adj5 (travel* or transport*)).kw. (103)
122 117 or 118 or 119 or 120 or 121 (3235)
123 Preparedness.kw,ti. (5095)
124 54 or 122 or 123 (1500903)
125 108 and 83 and 114 and 124 (980)
126 91 or 92 or 93 or 96 or 97 or 98 or 99 or 100 or 101 or 102 or 103 or 104 or 105 or 106 or 107 (159824)
127 126 and 83 and 114 and 124 (943)= setting focus + educatie + outcome + (cross border or preparedness or capacity building of international)

Psycinfo

Database: PsycINFO <1806 to September Week 4 2018>
Search Strategy:
----
1 exp public health/ (26716)
2 ((public adj3 health) or (communit* adj3 health)).tw. (67831)
3 ((public adj3 health) or (communit* adj3 health)).id. (25436)
4 preventive medicine/ (2032)
5 (preventive adj3 medicine).tw,id. (888)
6 ((infecti* adj3 prevent* adj3 contr*) or ipc).tw. (627)
7 ((infecti* adj3 prevent* adj3 contr*) or ipc).id. (40)
8 (surveill* adj3 transm* adj5 disease*).tw. (8)
9 (surveill* adj3 transm* adj5 disease*).id. (0)
10 pandemics/ (435)
11 (pandemic? or (disease adj3 outbreak?)).tw. (1985)
12 (pandemic? or (disease adj3 outbreak?)).id. (589)
13 disease transmission/ (1814)
14 (infect* adj3 disease* adj8 (transm* or manag*)).tw. (358)
15 (infect* adj3 disease* adj8 (transm* or manag*)).id. (55)
16 (infect* adj3 disease* adj8 (transm* or manag*)).tw. (358)
17 hazards/ (1602)
18 accidents/ or exp hazardous materials/ (9656)
19 occupational exposure/ or exp chemical exposure/ (3273)
20 radiation/ (1468)
21 emergency preparedness/ (1069)
22 emergency services/ or exp crisis intervention services/ or emergency management/ or natural disasters/ (14622)
23 ((radioactive or chemical) adj3 hazard?).tw,id. (75)
24 biohazard*.tw,id. (15)
25 or/1-24 (107997)
26 "onderdeel setting".ti. (0)
27 "onderdeel cross border".ti. (0)
28 exp transportation/ (7710)
29 immigration/ or exp human migration/ or refugees/ (29789)
30 exp aviation/ (2262)
31 (crossborder or "cross-border" or bordercross* or "border-cross" or "border crossing" or "border crossings" or "ground-cross" or "ground crossing" or "ground crossings" or groundcross* or "point of entry" or "points of entry" or transport or transfer or airport* or aeroport* or plane or port or ports or harbors or harbours or ship* or boat or boats or vessel * or craft* or vehicle* or car or cars or bus or coach or conveyanc*).tw. (106011)
32 (crossborder or "cross-border" or bordercross* or "border-cross" or "border crossing" or "border crossings" or "ground-cross" or "ground crossing" or "ground crossings" or groundcross* or "point of entry" or "points of entry" or transport or transfer or airport* or aeroport* or plane or port or ports or harbors or harbours or ship* or boat or boats or vessel * or craft* or vehicle* or car or cars or bus or coach or conveyanc*).id. (22239)
33 airport?.tw,id. (875)
34 (port adj5 health).tw,id. (21)
35 travel*.ti,id. (4204)
36 (border* or cross?border*).tw,id. (30776)
37 or/28-36 (171849)
38 25 and 37 (5660)

39 education/ or exp adult education/ or distance education/ or education policy/ or exp nontraditional education/ or nursing education/ or exp personnel training/ (63667)
40 continuing education/ or exp inservice training/ or adult learning/ or distance education/ or individualized instruction/ or professional development/ or exp training/ (94392)
41 (train* or supervis* or course* or hands?on or didacti* or exercis* or educat* or program* or skill* or curricul*).tw,id. (1227369)
42 evidence based practice/ or best practices/ or exp experimentation/ (94910)
43 (train* adj8 evaluat*).tw. (13906)
44 (pre?test* or post?test*).tw. (22182)
45 (educat* adj5 (result* or outreach or improv*)).tw. (25097)
46 ((transfer* adj3 skill*) or (improve adj3 skill*)).tw. (4964)
47 program evaluation/ or educational program evaluation/ (17642)
48 (program adj3 (evaluat* or effect* or appropiat*)).tw. (21908)
49 ((transfer* adj3 skill*) or (improve adj3 skill*)).tw. (4964)
50 (educat* adj5 (result* or outreach or improv*)).tw. (25097)
51 educational quality/ or course evaluation/ or educational program accreditation/ or educational program evaluation/ or exp educational standards/ or teacher effectiveness/ or teacher effectiveness evaluation/ (15013)
52 ((quality adj5 improv*) or (best adj3 practic*)).tw,id. (43212)
53 or/39-52 (1328574)
54 25 and 37 and 53 (2300)
55 (capacit* adj3 build*).tw. (3675)
56 (capacit* adj3 build*).id. (471)
57 international relations/ (3352)
58 international.ti. (15212)
59 international.id. (16478)
60 health/ (50246)
61 (population adj3 health).ti,id. (1038)
62 transm*.ti. (9404)
63 *disease transmission/ (1310)
64 or/55-63 (88403)
65 54 and 64 (204)
66 65 (204)
67 limit 66 to all journals (126)
68 *public health/ (14410)
69 ((public adj3 health) or (communit* adj3 health)).id,ti. (28189)
70 ((infecti* adj3 prevent* adj3 contr*) or ipc).id,ti. (62)
71 (surveill* adj3 transm* adj5 disease*).ti,id. (3)
72 *pandemics/ (320)
73 (pandemic? or (disease adj3 outbreak?)).ti,id. (695)
74 *disease transmission/ (1310)
75 (infect* adj3 disease* adj8 (transm* or manag*)).ti,id. (71)
76 or/68-75 (33413)
77 39 or 40 or 41 (1239589)
78 (test adj3 scor*).tw,id. (25725)
79 (emergenc* adj3 respon*).tw,id. (1562)
80 ((positive adj3 result*) or (deal* adj3 prepar*) or (cost-effic* adj3 train*)).tw,id. (14538)
81 42 or 43 or 44 or 45 or 46 or 47 or 48 or 49 or 50 or 51 or 52 or 78 or 79 or 80 (264482)
82 28 or 30 or 31 or 32 or 33 or 34 or 35 or 36 (143681)
83 55 or 56 or 57 or 58 or 59 (28164)
84 21 or 22 (15236)
85 preparedness.ti,id. (1652)

86 84 or 85 (16272)

87 76 and 77 and 81 and (82 or 83 or 86) (259)= setting focus+ outcome + (cross border or preparedness or capacity building of international) deze set wordt ingeperkt tot tijdschriftartikelen in set 89

88 87 (259)

89 limit 88 to all journals (189)

ERIC

Database: ERIC <1965 to April 2018>
Search Strategy:

----

1 exp public health/ (5400)
2 ((public adj3 health) or (communit* adj3 health)).tw. (11236)
3 (preventive adj3 medicine).tw. (952)
4 ((infecti* adj3 prevent* adj3 contr*) or ipc).tw. (66)
5 (surveill* adj3 transm* adj5 disease*).tw. (2)
6 exp communicable diseases/ (3874)
7 preventive medicine/ (887)
8 (pandemic? or (disease adj3 outbreak?)).tw. (258)
9 (infect* adj3 disease* adj8 (transm* or manag*)).tw. (53)
10 hazardous materials/ (1240)
11 radiation/ or exp laboratory safety/ (1783)
12 emergency programs/ or civil defense/ or crisis intervention/ or crisis management/ or emergency shelters/ or natural disasters/ (4756)
13 ((radioactive or chemical) adj3 hazard?).tw. (68)
14 biohazard*.tw. (12)
15 or/1-14 (22420)
16 "onderdeel setting".ti. (0)
17 "onderdeel cross border".ti. (0)
18 exp motor vehicles/ or exp transportation/ or exp travel/ (7000)
19 migration/ or immigrants/ or migrants/ or occupational mobility/ or refugees/ or undocumented immigrants/ (18551)
20 (crossborder or "cross-border" or bordercross* or "border-cross" or "border crossing" or "border crossings" or "ground-cross" or "ground crossing" or "ground crossings" or groundcross* or "point of entry" or "points of entry" or transport or transfer or airport* or aeroport* or plane or port or ports or harbors or harbours or ship* or boat or boats or vessel * or craft* or vehicle* or car or cars or bus or coach or conveyanc*).tw. (61332)
21 airport?.tw. (266)
22 (port adj5 health).tw. (1)
23 travel*.tw. (7745)
24 (border* or cross?border*).tw. (4382)
25 or/18-24 (91658)
26 (train* or supervis* or course* or hands?on or didacti* or exercis* or educat* or program* or skill* or curricul*).tw. (1389196)
27 evidence based practice/ (1656)
28 exp educational methods/ or exp education/ or exp educational practices/ or exp educational technology/ or nontraditional education/ (1072935)
29 staff development/ or exp labor force development/ or "coaching (performance)"/ or inservice education/ or on the job training/ or professional development/ or staff orientation/ or supervisor supervisee relationship/ or workplace learning/ (75164)
30 (train* adj8 evaluat*).tw. (12156)
31 (pre?test* or post?test*).tw. (27066)
32 (educat* adj5 (result* or outreach or improv*)).tw. (60513)
33 ((transfer* adj3 skill*) or (improve adj3 skill*)).tw. (5695)
34 program evaluation/ or course evaluation/ or curriculum evaluation/ or educational assessment/ or educational indicators/ or instructional material evaluation/ or outcome measures/ or "outcomes of education"/ or program attitudes/ or program effectiveness/ or program improvement/ or program validation/ or validated programs/ (153049)

35 (program adj3 (evaluat* or effect* or appropiat*)).tw. (106937)
36 ((transfer* adj3 skill*) or (improve adj3 skill*)).tw. (5695)
37 (educat* adj5 (result* or outreach or improv*)).tw. (60513)
38 educational quality/ or benchmarking/ or curriculum evaluation/ or educational improvement/ or educational objectives/ or program effectiveness/ or quality assurance/ or school effectiveness/ or teacher effectiveness/ (160095)
39 ((quality adj5 improv*) or (best adj3 practic*)).tw. (23335)
40 or/26-39 (1415708)
41 15 and 25 and 40 (1133)
42 capacity building/ (1814)
43 international cooperation/ or global approach/ or world problems/ (18689)
44 international.ti. (13081)
45 health/ (4833)
46 (disease adj3 transmi*).tw. (239)
47 (transm* adj5 prev*).tw. (284)
48 (capacit* adj3 build*).tw. (4549)
49 or/42-48 (38979)
50 41 and 49 (96)
51 exp *public health/ (505)
52 ((public adj3 health) or (communit* adj3 health)).ti. (1461)
53 ((infecti* adj3 prevent* adj3 contr*) or ipc).ti. (4)
54 (surveill* adj3 transm* adj5 disease*).ti. (0)
55 exp *communicable diseases/ (274)
56 (pandemic? or (disease adj3 outbreak?)).ti. (55)
57 (infect* adj3 disease* adj8 (transm* or manag*)).ti. (8)
58 or/51-57 (2154)
59 (test adj3 scor*).tw. (17919)
60 (emergenc* adj3 respon*).tw. (432)
61 ((positive adj3 result*) or (deal* adj3 prepar*) or (cost-effic* adj3 train*)).tw. (4298)
62 40 or 59 or 60 or 61 (1418102)
63 emergency programs/ (1631)
64 preparedness.tw. (2515)
65 58 and 62 and (25 or 42 or 48 or 43 or 44 or 63 or 64) (132) = setting focus+ outcome + (cross border or preparedness or capacity building of international)

Cinahl

Nb S35 met 221 refs is de slotset met)= setting focus + educatie + outcome + (cross border or preparedness or capacity building of international)

#	Query	Results
S35	S31 AND S34	221
S34	S32 OR S33	46,002
S33	(TI (crossborder or "cross-border" or bordercross* or "border-cross" or "border crossing" or "border crossings" or "ground-cross" or "ground crossing" or "ground crossings" or groundcross* or "point of entry" or "points of entry" or transport or transfer or airport* or aeroport* or plane or port or ports or harbors or harbours or ship* or boat or boats or vessel * or craft* or vehicle* or car or cars or bus or coach or conveyanc*)) OR (TI ((airport* or (port N5 health) or travel* or border* or cross*))	42,862
S32	TI(capacit* N3 build*) OR TI Preparedness OR (TI(international N5 (travel* or transport*)))	3,445
S31	S8 AND S20 AND S29 AND S30	1,051
S30	S13 OR S25 OR S26 OR S27	206,674
S29	S8 AND S20 AND S28	9,864
S28	S17 OR S18 OR S19 OR S21 OR S22 OR S23 OR S24	163,463
S27	TI Preparedness OR AB Preparedness OR SU Preparedness	4,728
S26	(TI(international N5 (travel* or transport*))) OR (AB(international N5 (travel* or transport*)).)	570
S25	TI(capacit* N3 build*) OR AB(capacit* N3 build*)	2,369
S24	TI(emergenc* N3 respon*) OR AB(emergenc* N3 respon*)	2,357
S23	(TI((positive N3 result*) or (deal* N3 prepar*) or (cost-effic* N3 train*))) OR (AB((positive N3 result*) or (deal* N3 prepar*) or (cost-effic* N3 train*)))	10,714
S22	TI(test N3 scor*) OR AB(test N3 scor*)	6,326
S21	(MH "Professional Practice, Evidence-Based+")	51,844
S20	S14 OR S15 OR S16	946,506
S19	(MH "Quality Improvement+")	39,507
S18	TI ((educat* N5 (result* or outreach or improv*))) OR AB ((educat* N5 (result* or outreach or improv*))) OR TI (((quality N5 improv*) or (best N3 practic*))) OR AB (((quality N5 improv*) or (best N3 practic*)))	67,630
S17	TI ((educat* N5 (result* or outreach or improv*))) OR AB ((educat* N5 (result* or outreach or improv*))) OR TI (((transfer* N3 skill*) or (improve N3 skill*))) OR AB (((transfer* N3 skill*) or (improve N3 skill*)))	19,065

S16	TI ((train* or supervis* or course* or hands?on or didacti* or exercis* or educat* or program* or skill* or curricul*)) OR AB ((train* or supervis* or course* or hands?on or didacti* or exercis* or educat* or program* or skill* or curricul*)) OR TI (train* N8 evaluat*) OR AB (train* N8 evaluat*)	575,556
S15	(MH "Public Health+/ED")	5,679
S14	(MH "Education+")	602,803
S13	S9 OR S10 OR S11 OR S12	200,352
S12	TI ((airport* or (port N5 health) or travel* or border* or cross*)) OR AB ((airport* or (port N5 health) or travel* or border* or cross*))	122,364
S11	TI ((crossborder or "cross-border" or bordercross* or "border-cross" or "border crossing" or "border crossings" or "ground-cross" or "ground crossing" or "ground crossings" or groundcross* or "point of entry" or "points of entry" or transport or transfer or airport* or aeroport* or plane or port or ports or harbors or harbours or ship* or boat or boats or vessel * or craft* or vehicle* or car or cars or bus or coach or conveyanc*)) OR AB ((crossborder or "cross-border" or bordercross* or "border-cross" or "border crossing" or "border crossings" or "ground-cross" or "ground crossing" or "ground crossings" or groundcross* or "point of entry" or "points of entry" or transport or transfer or airport* or aeroport* or plane or port or ports or harbors or harbours or ship* or boat or boats or vessel * or craft* or vehicle* or car or cars or bus or coach or conveyanc*))	65,637
S10	(MH "Transportation+")	13,217
S9	(MH "Travel+") OR (MH "Vehicle Operation+")	14,388
S8	S1 OR S2 OR S3 OR S4 OR S5 OR S6 OR S7	290,942
S7	TI ((infect* N3 disease*) N8 (transm* or manag*))	181
S6	TI(pandemic? or (disease N3 outbreak?))	271
S5	TI (surveill* N3 transm* N5 disease*)	13
S4	TI ((infecti* N3 prevent* N3 contr*) or ipc))	530
S3	TI ((public N3 health) or (communit* N3 health))	23,608
S2	(MM "Disease Outbreaks")	9,629
S1	(MM "Public Health+")	

Web of Science

28 September 2018

Search History					
**Set**	**Results**	**Save History / Create AlertOpen Saved History**	**Edit Sets**	**Combine Sets** AND OR **Combine**	**Delete Sets Select All Delete**
# 13	705	#12 AND #4<br>*Indexes=SCI-EXPANDED, SSCI, A&HCI, ESCI Timespan=All years*	Edit		
# 12	1,375	#11 AND #6<br>*Indexes=SCI-EXPANDED, SSCI, A&HCI, ESCI Timespan=All years*	Edit		
# 11	41,584	#10 OR #9<br>*Indexes=SCI-EXPANDED, SSCI, A&HCI, ESCI Timespan=All years*	Edit		
# 10	14,990	**TOPIC:** (preparedness)<br>*Indexes=SCI-EXPANDED, SSCI, A&HCI, ESCI Timespan=All years*	Edit		
# 9	26,896	**TOPIC:** (capacit* NEAR/3 build*) *OR* **TOPIC:** (international* NEAR/5 (travel* OR transport*)) *OR* **TOPIC:** (cross-border* OR (cross NEAR/5 border))<br>*Indexes=SCI-EXPANDED, SSCI, A&HCI, ESCI Timespan=All years*	Edit		

# 8	79	#7 AND #5<br>*Indexes=SCI-EXPANDED, SSCI, A&HCI, ESCI Timespan=All years*	Edit	☐	☐
# 7	17,741	#6 AND #4<br>*Indexes=SCI-EXPANDED, SSCI, A&HCI, ESCI Timespan=All years*	Edit	☐	☐
# 6	79,452	#3 OR #2 OR #1<br>*Indexes=SCI-EXPANDED, SSCI, A&HCI, ESCI Timespan=All years*	Edit	☐	☐
# 5	10,737	**TOPIC:** (TOPIC: ((evidenc* NEAR/3 based) or (train* NEAR/5 evaluat*) or pretest* or posttest*) OR TOPIC: (educat* NEAR/5 (result* or outreach or improv*)) OR TOPIC: ((quality NEAR/2 (manag* or improv*))) OR TOPIC: (best NEAR/3 practic*))<br>*Indexes=SCI-EXPANDED, SSCI, A&HCI, ESCI Timespan=All years*	Edit	☐	☐
# 4	3,496,087	**TOPIC:** (((train* or supervis* or course* or hands* or didacti* or exercis* or educat* or program* or skill* or curricul*)) OR TOPIC: (((transfer* NEAR/3 skill*) or (improve NEAR/3 skill*))))<br>*Indexes=SCI-EXPANDED, SSCI, A&HCI, ESCI Timespan=All years*	Edit	☐	☐
# 3	251	**TITLE:** (pandemic* NEAR/3 transm*) *OR* **TITLE:** (pandemic* NEAR/3 outbreak*)<br>*Indexes=SCI-EXPANDED, SSCI, A&HCI, ESCI Timespan=All years*	Edit	☐	☐
# 2	9,633	**TITLE:** (surveill* NEAR/3 transm*) *OR* **TITLE:** (disease NEAR/3 outbreak*) *OR* **TITLE:** (infect* NEAR/3 transm*) *OR* **TITLE:** (iinfect* NEAR/3 manag*)	Edit	☐	☐

		Indexes=SCI-EXPANDED, SSCI, A&HCI, ESCI Timespan=All years			
# 1	70,061	**TITLE:** (public NEAR/3 health) *OR* **TITLE:** (communit* NEAR/3 health) *OR* **TITLE:** (infecti* NEAR/3 prevent*) *OR* **TITLE:** (infecti* NEAR/3 control*)<br>*Indexes=SCI-EXPANDED, SSCI, A&HCI, ESCI Timespan=All years*	Edit		
				AND OR<br>**Combine**	**Select All Delete**
