# Supplementary material for: Educating, training, and exercising for infectious disease control with emphasis on cross-border settings: an integrative review
Source: Global Health. 2020 Sep 3;16:78. doi: 10.1186/s12992-020-00604-0 (PMC7468091; doi:10.1186/s12992-020-00604-0)
Supplement: Supplementary file 3 — Additional file 3. Quality Assessment form. The form composed for quality assessment of the studies; the first part assessing the quality of the education, training or exercise method and the second part that of the performed scientific study. [file 12992_2020_604_MOESM3_ESM.pdf]

Additional file 2 – Quality Assessment form

Quality Assessment - Article: ………………………………………….

(Yes = 2 points; Partly = 1 point; No / Unclear = 0 points; ETE = education, training, and exercise).

Good = ≥2/3 of max score, moderate = 1/3 ≥ max score >2/3, bad = < 1/3 score

Part 1 - Training (Based on theYouth Department Council of Europe. Quality standards in education and training activities of the Youth Department of the Council of Europe. DDCP-YD/ETD (2016); 202.) **:**

1. A needs assessment among the target population is conducted before the ETE.

   Yes / Partly / No / Unclear, Comments: …………………………………..
2. Concrete, achievable and assessable ETE objectives are stated.

   Yes / Partly / No / Unclear, Comments: …………………………………..
3. A competent team of trainers and facilitators guided the training (Content and facilitating experience).

   Yes / Partly / No / Unclear, Comments: …………………………………..
4. Adequate recruitment and selection of participants took place (representatives, motivated).

   Yes / Partly / No / Unclear, Comments: …………………………………..
5. A clear description of training methods is provided (what, where, how, why).

   Yes / Partly / No / Unclear, Comments: …………………………………..
6. A thorough process of evaluation has been conducted. (Pre-, post-, and follow-up test > 1 month, Partly = two of the three tests.)

   Yes / Partly / No / Unclear, Comments: …………………………………..

   Endscore training: …………………………………… Label: good / moderate / bad

Part 2 - Study (Based on the National Institute for Health and Care Excellence (NICE). Methods for the development of NICE public health guidance (thrid edition). NICE 2012.) **:**

7. The context of the training is clearly described (domain and setting (local/regional/national)).
   Yes / Partly / No / Unclear, Comments: …………………………………….
8. The research design is defensible rigorous (precision of planning, data collection, analysis, and reporting).
   Yes / Partly / No / Unclear, Comments: …………………………………….
9. The analysis valid (does it measure what it aims to measure?).
   Yes / Partly / No / Unclear, Comments: …………………………………….
10. The findings are convincing & relevant to the aims of the study (Yes = both, Partly = 1 of 2 or both partly).
    Yes / Partly / No / Unclear, Comments: …………………………………….
11. Conclusions are plausible and cohorent (Yes = both, Partly = 1 of 2 or both partly).
    Yes / Partly / No / Unclear, Comments: …………………………………….
12. Implications are stated, and limitations encountered. (Yes = both, Partly = 1 of 2 or both partly)
    Yes / Partly / No / Unclear, Comments: …………………………………….

    Endscore Study: …………………………………….Label: good / moderate / bad
