# Supplementary material for: Educating, training, and exercising for infectious disease control with emphasis on cross-border settings: an integrative review
Source: Global Health. 2020 Sep 3;16:78. doi: 10.1186/s12992-020-00604-0 (PMC7468091; doi:10.1186/s12992-020-00604-0)
Supplement: Supplementary file 4 — Additional file 4. Results Quality Assessment. The results of the quality assessment are shown here. [file 12992_2020_604_MOESM4_ESM.pdf]

Additional file 3 – Results Quality Assessment

**Study** (First author, year)	**Training quality Score** (0-12)	**Training quality** (Good/Moderate/Bad)	**Study quality Score** (0-12**)**	**Study Quality** (Good/Moderate/Bad)	**Total score** (0-24)	**Total quality** (Good/Moderate/Bad)
Ablah E, 2007	6	moderate	9	good	15	moderate
Ablah E, 2008,	7	moderate	12	good	19	good
Aiello A., 2011	6	moderate	10	good	16	moderate
Alexander, 2005	5	moderate	3	bad	8	bad
Alexander, 2008	9	good	9	good	18	good
Araz O, 2012	4	bad	4	bad	8	bad
Araz OM, 2013	7	moderate	7	moderate	14	moderate
Atack L, 2008	3	bad	8	moderate	11	moderate
Atlas RM, 2005	4	bad	3	bad	7	
Baldwin K, 2005	6	moderate	3	bad	9	moderate
Bazeyo W, 2015	5	Moderate	3	bad	8	bad
Becker KM, 2012	6	moderate	6	moderate	12	moderate
Berrian AM, 2018	4	bad	10	good	14	moderate
Biddinger PD, 2010	5	moderate	9	good	14	moderate
Cathcart LA, 2018	5	moderate	7	moderate	12	moderate
Chandler T, 2008	5	moderate	7	moderate	12	moderate

Chiu M, 2011	4	bad	7	moderate	11	moderate
Craig AT, 2007	6	moderate	6	moderate	12	moderate
Dausey DJ, 2007	2	bad	10	good	12	moderate
Dausey DJ, 2014	6	moderate	3	bad	9	moderate
Dickmann P, 2016	5	moderate	3	bad	8	moderate
El-Bahnasawy MM, 2014	1	bad	1	bad	2	bad
Faass J, 2013	3	bad	3	bad	6	bad
Fowkes V, 2007	9	good	8	moderate	17	good
Fowkes V, 2010	10	good	10	good	20	good
Gershon RR, 2009	7	moderate	8	moderate	15	moderate
Grillo M, 2017	4	bad	8	Moderate	12	moderate
Hegle J, 2011	6	moderate	7	moderate	13	moderate
Hoeppner MM, 2010	9	good	9	good	18	good
Horney JA, 2005	7	moderate	6	moderate	13	moderate
Hueston WD, 2008	6	moderate	4	bad	10	moderate
Johnson YF, 2009	5	moderate	8	moderate	13	moderate
Kohn S, 2010	5	moderate	7	moderate	12	moderate

Livet M, 2005	5	moderate	9	good	14	moderate
Macario E, 2007	5	moderate	7	moderate	12	moderate
Martin G, 2018	4	bad	4	bad	8	bad
Mitka M, 2003	2	bad	4	bad	6	bad
Morris JG, 2012	5	moderate	6	moderate	11	moderate
Olson D, 2008	8	moderate	4	bad	12	moderate
Orfaly RA, 2005	6	moderate	6	moderate	12	moderate
Orfaly RA, 2005a	8	good	4	bad	12	moderate
Otto JL, 2010	7	moderate	7	moderate	14	moderate
Peddecord KM, 2007	5	moderate	10	good	15	moderate
Potter MA, 2005	6	moderate	9	good	15	moderate
Quiram BJ, 2005	6	moderate	4	bad	10	moderate
Qureshi, KA, 2004	7	moderate	11	good	18	good
Rega PP, 2014	5	moderate	7	moderate	12	moderate
Richter J, 2005	4	bad	11	good	15	moderate
Rottman, SJ, 2005	7	moderate	8	moderate	15	moderate
Sandstrom, BE, 2014,	6	moderate	10	good	16	moderate
Sarpy SA, 2005	11	good	10	good	21	good
Savoia E, 2009	6	moderate	10	good	16	moderate
Savoia E, 2013	7	moderate	9	good	16	moderate
Soeters HM, 2018	3	bad	12	good	15	moderate
Taylor JL, 2005	5	moderate	4	bad	9	moderate

Umble KE, 2000	7	moderate	12	good	19	good
Waltz EC, 2010	4	bad	8	moderate	12	moderate
Wang C, 2008	10	good	11	good	21	good
Wang C, 2008a	6	moderate	9	good	15	moderate
Wang C, 2010	11	good	11	good	22	good
Yamada S, 2007	4	bad	6	moderate	10	moderate
Yellowlees P, 2008	6	moderate	6	moderate	12	moderate
